# Cataract Development Among Pediatric Patients With Uveitis

**DOI:** 10.1001/jamanetworkopen.2024.19366

**Published:** 2024-07-01

**Authors:** Alan Y. Hsu, Hou-Ting Kuo, Chun-Ju Lin, Ning-Yi Hsia, Shu-Chun Kuo, Chang-Ching Wei, Chun-Ting Lai, Huan-Sheng Chen, Yu-Hsun Wang, James Cheng-Chung Wei, Yi-Yu Tsai

**Affiliations:** 1 Department of Ophthalmology, China Medical University Hospital, China Medical University, Taichung City, Taiwan; 2 Department of General Medicine, China Medical University Hospital, Taifchung City, Taiwan; 3 School of Medicine, College of Medicine, China Medical University, Taichung City, Taiwan; 4 Department of Optometry, Asia University, Taichung City, Taiwan; 5 Department of Ophthalmology, Chi Mei Medical Center, Tainan City, Taiwan; 6 Department of Optometry, Chung Hwa University of Medical Technology, Tainan City, Taiwan; 7 Division of Allergy, Immunology and Rheumatology, Department of Pediatrics, Children’s Hospital, China Medical University Hospital, Taichung City, Taiwan; 8 An-Shin Dialysis Center, NephroCare Ltd, Fresenius Medical Care, Taichung City, Taiwan; 9 Department of Medical Research, Chung Shan Medical University Hospital, Taichung City, Taiwan; 10 Institute of Medicine, Chung Shan Medical University, Taichung City, Taiwan; 11 Department of Allergy, Immunology and Rheumatology, Chung Shan Medical University Hospital, Taichung City, Taiwan; 12 Institute of Integrated Medicine, China Medical University, Taichung City, Taiwan

## Abstract

**Importance:**

The long-term estimated risk of development of cataracts among pediatric patients with uveitis is not clear.

**Objective:**

To describe factors associated with the development of cataracts among pediatric patients with uveitis.

**Design, Setting, and Participants:**

This cohort study used the international TriNetX database to enroll pediatric patients with and without uveitis from January 1, 2002, to December 31, 2022. The nonuveitis cohort consisted of randomly selected control patients matched by age, sex, race and ethnicity, and specific comorbidities.

**Exposure:**

Diagnosis of uveitis, identified using diagnostic codes.

**Main Outcomes and Measures:**

The primary outcome was the risk of developing cataracts among the uveitis group compared with the nonuveitis comparison group, with hazard ratios (HRs) and 95% CIs reported.

**Results:**

A total of 22 687 pediatric patients with uveitis (mean [SD] age, 10.3 [5.6] years; 54.2% male) and 22 687 comparators without uveitis (mean [SD] age, 10.3 [5.6] years; 54.5% male) were enrolled in the study. The risk of cataracts was increased among pediatric patients with uveitis up to a follow-up duration of 20 years (HR, 17.17; 95%CI, 12.90-22.80) from the index date. Subgroup analyses revealed an elevated cataract risk across age groups: 0 to 6 years (HR, 19.09; 95% CI, 10.10-36.00), 7 to 12 years (HR, 27.16; 95% CI, 15.59-47.20), and 13 to 18 years (HR, 13.39; 95% CI, 8.84-20.30); both female sex (HR, 13.76; 95% CI, 9.60-19.71) and male sex (HR, 11.97; 95% CI, 8.47-16.91); and Asian (HR, 13.80; 95% CI, 3.28-58.07), Black or African American (HR, 10.41; 95% CI, 5.60-19.36), and White (HR, 15.82; 95% CI, 11.05-22.60) race. Furthermore, increased cataract risks were also observed among those with and without a history of immunosuppressive agents (with: HR, 26.52 [95% CI, 16.75-41.90]; without: HR, 17.69 [95% CI: 11.39-27.40]), a history of steroid eye drop use (with: HR, 29.51 [95% CI, 14.56-59.70]; without: HR, 16.49 [95% CI, 11.92-22.70]), and a history of intraocular procedures (with: HR, 11.07 [95%CI, 4.42-27.71]; without: HR, 14.49 [95% CI, 10.11-20.70]).

**Conclusions and Relevance:**

In this cohort study of pediatric patients with uveitis, an elevated risk of cataracts following a uveitis diagnosis was found compared with pediatric patients without uveitis. The findings suggest that pediatric patients with uveitis should be monitored for cataract development.

## Key Points

**Question:**

What are the prevalence of and risk factors associated with the development of cataracts in patients younger than 18 years with uveitis?

**Findings:**

In this cohort study of 22 687 pediatric patients in the US younger than 18 years with uveitis, a significantly elevated risk of cataracts was observed compared with 22 687 propensity-matched patients younger than 18 years without uveitis. Furthermore, this association persisted from the index date to a follow-up duration of up to 20 years.

**Meaning:**

The findings of this study suggest that pediatric patients with uveitis should be monitored for cataract development.

## Teaser

This cohort study compares pediatric patients with and without uveitis to assess whether a uveitis diagnosis is associated with cataract development.

## Introduction

Pediatric cataracts are a significant cause of childhood blindness globally.^1^ A better understanding of the etiologies for pediatric cataracts is crucial for ensuring optimal clinical outcomes for these vulnerable patients. Uveitis is one of the most common ophthalmologic conditions affecting pediatric eyes and is known to be associated with an increased risk for cataract formation.^2,3^ Other factors that could contribute to this risk for cataract formation include the use of steroid eye drops and other immunosuppressive medications commonly prescribed for uveitis management. While the exact mechanisms remain unknown, certain animal models have demonstrated that immunosuppressive agents can adversely affect the growth of lens epithelial cells, potentially leading to cataract formation.^4^ Additionally, intraocular surgeries could lead to a postoperative increase in inflammation within the vitreous.^5^ Such a postoperative increase in inflammation could then potentially cause damage to lens proteins through oxidative stress. Although inflammation may be one of the important links between uveitis and cataract formation, the actual pathophysiological mechanism behind cataract development among patients younger than 18 years with uveitis remains unknown. However, as pediatric patients are known to have immature blood-ocular barriers,^6^ it is possible that this immaturity may play a crucial role in initiating or perpetuating intraocular inflammatory reactions, subsequently leading to cataracts. This is because the eye is normally an immune-privileged site due to the blood-ocular barrier. However, when this barrier is disrupted, it can allow infiltration of inflammatory cells, leading to intraocular inflammatory reactions or uveitis. This has potential implications for future cataract development, supported by previous laboratory studies suggesting that oxidative stress may be integral to cataract formation.^7^ For example, Lesiewska et al^8^ demonstrated higher concentrations of oxidative products like superoxide dismutase among cataract eyes compared with noncataract eyes. It is also possible that the combination of other factors, such as inherited pro-cataract mutations in the *SOX2* gene, could further increase the susceptibility of patients younger than 18 years in developing cataracts, especially after uveitis (eFigure 5 in Supplement 1). However, these hypotheses remain speculative, and as of now, no study, to our knowledge, has definitively established a conclusive association between all of these mediating factors in the context of uveitis and cataract development.

In terms of the literature, there have been reports on the increased risk of cataract development in patients with uveitis, as summarized in eTable 14 in Supplement 1. However, many existing studies are limited to being single institutional, having small sample sizes, or lacking a specific focus on the pediatric population. For instance, Minkus et al^2^ conducted a single-institutional retrospective study with 2190 patients, suggesting an increased risk of cataracts among patients with uveitis, with a cumulative incidence rate of 36.6% by 10 years. Despite being one of the larger studies in the literature, it did not exclusively focus on pediatric age groups, leaving uncertainties about how such cataract risks translate to the pediatric population with uveitis. Similarly, larger studies by Blum-Hareuveni et al,^9^ AlBloushi et al,^10^ and Al-Ani et al^11^ that investigated cataract risk among patients with uveitis, also did not restrict their study populations to the pediatric age groups. While these studies support the association between uveitis and cataract formation, they lack specific demonstrations within the pediatric population in a multi-institutional context with rigorous study methods. Therefore, to address these knowledge deficits, we conducted a multi-institutional retrospective cohort study involving pediatric patients with uveitis to assess the risk of cataract formation.

## Methods

### Data Source and Study Design

This multi-institutional retrospective cohort study used the TriNetX analytics platform. This federated, cloud-based international health research platform contains up to 100 million deidentified patient records from 77 health care organizations across 9 countries. These organizations are part of regional collaborative networks, including those from the US; Europe, the Middle East, and Africa (EMEA); Latin America; and the Asia-Pacific regions. Specifically, our study primarily used the US research network within TriNetX, which encompasses data from 54 health care organizations across all 50 individual states in the US, totaling approximately 92 million patients. We also used the EMEA network to perform additional sensitivity analysis. Furthermore, any output from the TriNetX platform can only be displayed in aggregate counts and statistical summaries in deidentified forms. Another point to highlight is that TriNetX is not an open-access platform, and access is limited to researchers affiliated with tertiary medical institutions that have established agreements with TriNetX. The validity of using TriNetX for epidemiological studies has been established in prior research.^12^ The study adhered to the tenets of the Declaration of Helsinki.^13^ Because the study retrospectively used deidentified data from either database, the Chung Shan Medical University Hospital Institutional Review Board waived the patient consent requirement, and the Chung Shan Medical University Hospital Institutional Review Board approved the study. This study followed the Strengthening the Reporting of Observational Studies in Epidemiology (STROBE) reporting guideline.

For our study, the data extracted from the platform included basic demographic information, medical diagnoses (using the *International Statistical Classification of Diseases, Tenth Revision, Clinical Modification* [*ICD-10-CM*] codes) (Table 1), procedures (using the Current Procedural Terminology codes) (eTable 1 in Supplement 1), and medications (using the RxNorm codes) (eTable 2 in Supplement 1). Race and ethnicity–related data that were included in our study as potential covariates were reported by the health care institutions collaborating with the TriNetX platform. The TriNetX database is compliant with the International Organization for Standardization and with the Health Insurance Portability and Accountability Act.

**Table 1.** *ICD-10-CM* Codes Used for Enrollment and Outcome

*ICD-10-CM* code	Content
**Inclusion**	
Uveitis	
H16.24	Ophthalmia nodosa
H20	Iridocyclitis
H30	Pars planitis
H31	Disorders of the choroid
H35.06	Retinal vasculitis
H35.33	Angioid streaks of macula
H44.00	Panuveitis
H44.13	Sympathetic uveitis
Comorbidities	
J45	Asthma
L20	Atopic dermatitis
M05	Rheumatoid arthritis
M06	Other rheumatoid arthritis
M07	Enteropathic arthropathies
M08	Juvenile arthritis
K50	Crohn disease
K51	Ulcerative colitis
K90.00	Celiac disease
H52.1	Myopia
H52.0	Hypermetropia
Laboratory tests^a^	
TriNetX: 9063	C-Reactive protein level (mass/volume) in serum, plasma, or blood (mg/dL)
TriNetX: 9015	Leukocyte count (mean [SD]) in blood (/μL)
**Exclusion**	
B15-19	Viral hepatis
B20, B97.35, R75, Z21	HIV disease
E08-E13	Diabetes
A15-19	Tuberculosis
Q12.0	Congenital cataract
**Outcome**	
H26.0	Infantile, juvenile, and presenile cataract
H26.1	Traumatic cataract
H26.2	Complicated cataract
H26.3	Drug-induced cataract
H26.4	After cataract (secondary cataract)
H26.8	Other unspecified cataract
H26.9	Cataract unspecified
H27.8	Other disorders of lens
H27.8	Cataract in diseases classified elsewhere

Abbreviation: *ICD-10-CM*, *International Statistical Classification of Diseases, Tenth Revision, Clinical Modification*.

SI conversion factors: To convert C-reactive protein level to milligrams per liter, multiply by 10; to convert leukocyte count to ×10^9^ per liter, multiply by 0.001.

^a^ Laboratory codes were based on the Logical Observation Identifiers Names and Codes, which includes standardized codes used for such test results.

As part of our external validation analysis, we used Taiwan’s Longitudinal Health Insurance Database (LHID), a subset of the Taiwan National Health Insurance Research Database (NHIRD). The LHID and the NHIRD are federated cloud-based databases managed by the Taiwanese Bureau of National Health Insurance. The LHID comprises a randomized sample of over 1 million patients from the NHIRD collected since 2000. The NHIRD contains deidentified electronic diagnostic records from various health care institutions in Taiwan, covering more than 99% of the Taiwanese population. It includes detailed anonymized personal information such as sex, age, and diagnoses and treatments from outpatient and inpatient settings. Clinical information in the NHIRD is classified using the *ICD-10-CM* codes. The NHIRD adheres to all relevant privacy laws and regulations of Taiwan, including the Taiwanese Personal Data Protection Act. Access to this database is restricted to researchers affiliated with medical centers and institutions that have established agreements with the NHIRD. This database has been used in previous epidemiological studies.^14^

### Study Participants, Main Measures, and Outcomes

**Figure 1.**
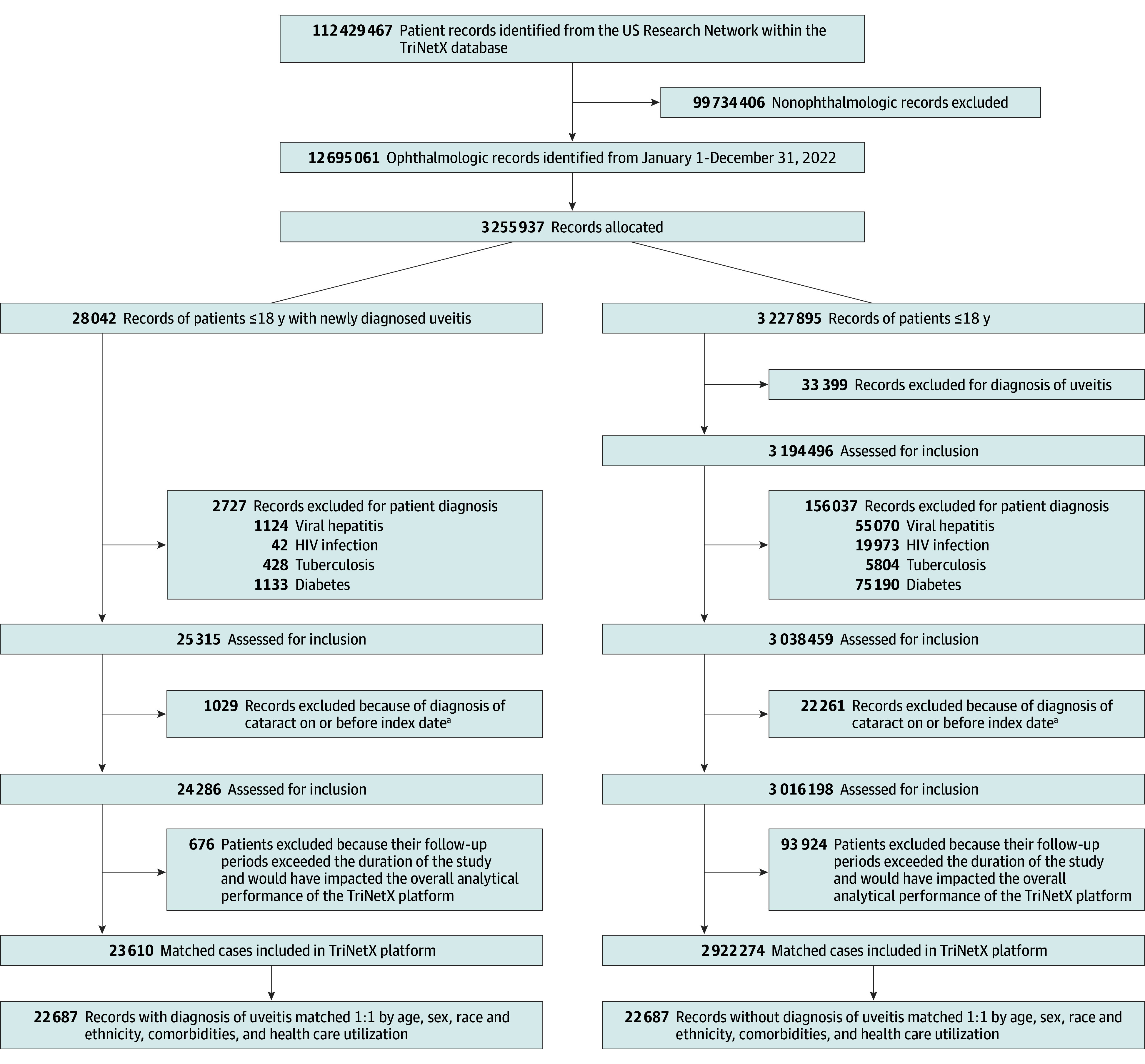
Patient Flow Diagram ^a^Cataract diagnosis Q12.0, H25-H28 based on the *International Statistical Classification of Diseases, Tenth Revision, Clinical Modification*.

Figure 1 and eFigure 1 in Supplement 1 depict our cohort construction. All patients 18 years or younger diagnosed with uveitis from January 1, 2002, to December 31, 2022, were enrolled as our study participants. The index event for the uveitis cohort was an encounter diagnosis based on the presence of *ICD-10-CM* diagnostic codes (Table 1).

Those 18 years or younger never diagnosed with uveitis were the comparison cohort. The comparison patients were randomly sampled from all registered patients in the TriNetX database who were excluded from our study and did not have uveitis. The date of the first event of uveitis was considered the uveitis index date. The comparison group’s index date was derived from a randomized date between January 1 and December 31, 2022, based on the first health care encounter with an ophthalmologist.

Each participant in our matched cohorts had their data censored when 1 of 2 scenarios was met: (1) the day after the last recorded data point within the specified time interval or when there was a delay between the index event and the start of the time window or (2) the patient developed the outcome of interest after the index event but prior to the start of the time window (the patient would then be censored at the beginning of the time window). To minimize confounding, the exclusion criteria (Table 1) were those with a history of viral hepatitis, HIV infection, diabetes, tuberculosis, and congenital cataracts.

Baseline clinical information and comorbidities were obtained 2 years before the index date. Baseline clinical information of interest included sex, age, race and ethnicity, and the setting of medical utilization. A 1:1 cohort was also created, with patients with uveitis precisely matched to individuals from the nonuveitis comparison group based on specific variables of interest. The variables considered in the propensity score–matching analysis included race (American Indian or Alaska Native, Asian, Black or African American, Native Hawaiian or Other Pacific Islander, White, other [including mixed race], and unknown race), ethnicity (Hispanic or Latino, non-Hispanic or non-Latino, and unknown ethnicity), sex, age, comorbidities of interest, the health care encounter’s setting, and laboratory data. Laboratory tests analyzed in this study included leukocyte count and C-reactive protein (CRP). The matching process was conducted using the built-in function from the TriNetX platform. All included data on race and ethnicity were reported by the health care organizations in partnership with the TriNetX platform. Race and ethnicity data were obtained because such factors are potential covariates in our study. The primary end point of this study was the first-time encounter diagnosis *ICD-10-CM* code of cataracts (Table 1) after the index date.

### Statistical Analysis

Our main study’s statistical analysis was performed using the TriNetX platform. We also performed external validation analyses using NHIRD data. To analyze the balance of distribution from among our baseline variables, standardized mean differences (SMDs) were used. Well-matched SMD values were defined as less than 0.1. For the analysis of the different variables within the matched cohort, we used the Cox proportional hazards regression analysis. Additional analyses, including positive outcome control and negative outcome control, were performed. These analyses were reported with hazard ratios (HRs) and 95% CIs. A log-rank test and the associated Kaplan-Meier method were also used to evaluate the incidence of cataracts. Statistical significance was defined as a 2-sided *P* < .05. Data were analyzed using the built-in functions of the TriNetX platform, which is a custom-developed system with many proprietary functions However, the known software packages and languages used by TriNetX include Java, version 11.0.16 (Oracle Corp); R, version 4.0.2 (R Project for Statistical Computing); and Python, version 3.7 (Python Software Foundation).

## Results

### Baseline Demographic and Clinical Characteristics

 A total of 22 687 pediatric patients with uveitis (mean [SD] age, 10.3 [5.6] years; 45.3% female, 54.2% male, and 0.5% unknown) and 22 687 comparators without uveitis (mean [SD] age, 10.3 [5.6] years; 45.0% female, 54.5% male, and 0.4% unknown) were enrolled for this study (Table 2). Among the patients with uveitis, the mean (SD) of CRP levels was 2.32 (4.35) mg/dL, and 2.03 (3.82) mg/dL among those without uveitis (to convert to milligrams per liter, multiply by 10.0). Among the patients with uveitis, the mean (SD) of serum leukocyte count was 9100 (43 100)/μL, and those without uveitis was 8600 (10 800)/μL (to convert to ×10^9^ per liter, multiply by 0.001). The information of interest obtained at baseline from both groups included demographics (eg, age, race and ethnicity, and sex), comorbidities (eg, asthma, atopic dermatitis, various arthritis conditions, inflammatory bowel disease, celiac disease, myopia, and hypermetropia), the settings of the health care encounters, and laboratory data. Of the total patients, there were 101 American Indian or Alaska Native patients with uveitis (0.4%) and 89 without (0.4%), 619 Asian patients with uveitis (2.7%) and 625 without (2.8%), 4409 Black or African American patients with uveitis (19.4%) and 4422 without (19.5%), 3727 Hispanic or Latino patients with uveitis (16.4%) and 3752 without (16.5%), 59 Native Hawaiian or Other Pacific Islander patients with uveitis (0.3%) and 58 without (0.3%), 15 013 non-Hispanic or non-Latino patients with uveitis (66.2%) and 15 024 without (66.2%), 12 358 White patients with uveitis (54.5%) and 12 411 without (54.7%), 1667 patients categorized as being other race with uveitis (7.3%) and 1647 without (7.3%), 3474 patients of unknown race with uveitis (15.3%) and 3435 without (15.1%), and 3947 patients of unknown ethnicity with uveitis (17.4%) and 3911 without (17.2%). These details were obtained before and after propensity score matching. The 2 groups were well matched in distribution across these baseline factors of interest, as indicated by SMDs less than 0.1.

**Table 2.** Demographic Characteristics of Patients With and Without Uveitis^a^

Characteristic	Patients before PSM, No. (%)		*P* value	SMD	Patients after PSM, No. (%)		*P* value	SMD
	With uveitis (n = 23 610)	Without uveitis (n = 2 922 274)			With uveitis (n = 22 687)	Without uveitis (n = 22 687)		
Age, mean (SD), y	10.3 (5.6)	6.6 (5.6)	<.001	0.67	10.3 (5.6)	10.3 (5.6)	.34	0.01
Sex								
Female	10 744 (45.5)	1 426 882 (48.8)	<.001	0.07	10 283 (45.3)	10 217 (45.0)	.53	0.01
Male	12 761 (54.0)	1 472 576 (50.4)	<.001	0.07	12 301 (54.2)	12 369 (54.5)	.52	0.01
Unknown	105 (0.4)	22 816 (0.8)	<.001	0.04	103 (0.5)	101 (0.4)	.89	0.001
Race								
American Indian or Alaska Native	108 (0.5)	13 998 (0.5)	.63	0.003	101 (0.4)	89 (0.4)	.38	0.01
Asian	658 (2.8)	105 887 (3.6)	<.001	0.05	619 (2.7)	625 (2.8)	.86	0.002
Black or African American	4554 (19.3)	526 363 (18.0)	<.001	0.03	4409 (19.4)	4422 (19.5)	.88	0.001
Native Hawaiian or Other Pacific Islander	60 (0.3)	8685 (0.3)	.23	0.01	59 (0.3)	58 (0.3)	.93	0.001
White	12 918 (54.7)	1 566 753 (53.6)	.001	0.02	12 358 (54.5)	12 411 (54.7)	.62	0.01
Other^a^	1735 (7.3)	235 098 (8.0)	<.001	0.03	1667 (7.3)	1647 (7.3)	.72	0.003
Unknown	3577 (15.2)	465 490 (15.9)	.001	0.02	3474 (15.3)	3435 (15.1)	.61	0.01
Ethnicity								
Hispanic or Latino	3864 (16.4)	556 700 (19.1)	<.001	0.07	3727 (16.4)	3752 (16.5)	.75	0.003
Non-Hispanic or non-Latino	15 688 (66.4)	1 888 144 (64.6)	<.001	0.04	15 013 (66.2)	15 024 (66.2)	.91	0.001
Unknown	4058 (17.2)	477 430 (16.3)	<.001	0.02	3947 (17.4)	3911 (17.2)	.66	0.004
Comorbidities								
Asthma	1375 (5.8)	180 596 (6.2)	.02	0.02	1277 (5.6)	1343 (5.9)	.18	0.01
Atopic dermatitis	460 (1.9)	88 751 (3.0)	<.001	0.07	432 (1.9)	418 (1.8)	.63	0.01
Rheumatoid arthritis^b^	10 (<0.1)	43 (<0.1)	<.001	0.03	10 (<0.1)	10 (<0.1)	1.00	<0.001
Other rheumatoid arthritis	178 (0.8)	707 (<0.1)	<.001	0.12	150 (0.7)	103 (0.5)	.003	0.03
Enteropathic arthropathies	10 (<0.1)	31 (<0.1)	<.001	0.03	10 (<0.1)	10 (<0.1)	1.00	<0.001
Juvenile arthritis	1229 (5.2)	2878 (0.1)	<.001	0.32	1137 (5.0)	1066 (4.7)	.12	0.02
Crohn disease (regional enteritis)	306 (1.3)	2152 (0.1)	<.001	0.15	302 (1.3)	364 (1.6)	.02	0.02
Ulcerative colitis	187 (0.8)	1142 (<0.1)	<.001	0.12	181 (0.8)	193 (0.9)	.53	0.01
Celiac disease	63 (0.3)	2067 (0.1)	<.001	0.05	57 (0.3)	36 (0.2)	.03	0.02
Myopia	582 (2.5)	0	<.001	0.22	0	0	NA	NA
Hypermetropia	371 (1.6)	0	<.001	0.18	0	0	NA	NA
Medical utilization								
Ambulatory	12 876 (54.54)	1 735 160 (59.38)	<.001	0.10	11 974 (52.78)	11 971 (52.77)	.98	<0.001
Emergency	4963 (21.02)	546 540 (18.70)	<.001	0.06	4699 (20.71)	4803 (21.17)	.23	0.01
Inpatient encounter	1723 (7.30)	168 120 (5.75)	<.001	0.06	1602 (7.06)	1654 (7.29)	.34	0.01
Laboratory results, mean (SD)								
C-Reactive protein level in serum, plasma, or blood, mg/dL	2.25 (4.24)	2.36 (4.11)	.34	0.03	2.32 (4.35)	2.03 (3.82)	.08	0.07
Leukocyte count in blood, /μL	9000 (41 600)	9200 (28 700)	.67	0.01	9100 (43 100)	8600 (10 800)	.48	0.02

Abbreviations: NA, not applicable; PSM, propensity score matching; SMD, standardized mean difference.

SI conversion factors: To convert C-reactive protein level to milligrams per liter, multiply by 10; leukocyte count to ×10^9^ per liter, multiply by 0.001.

^a^ Includes mixed race.

^b^ With a rheumatoid factor.

### Risk of Cataracts in Terms of Follow-Up Duration

**Figure 2.**
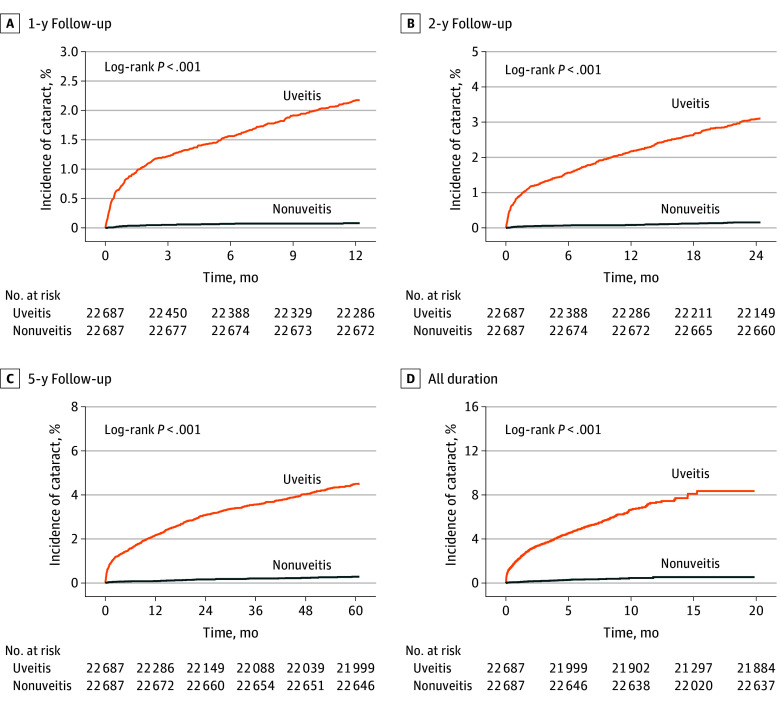
Kaplan-Meier Analysis for Risk of Cataract by Assessment Time See Table 3 for hazard ratios (95% CIs) for risk of cataracts among patients with uveitis compared with those without uveitis.

The primary outcome of this study was the risk of developing cataracts among the uveitis group compared with the nonuveitis group. The risk for cataract development was assessed at a specific follow-up duration of 1 year, 2 years, 5 years, and 20 years following the index date (Table 3 and Figure 2).

**Table 3.** Risk of Cataract in Patients With Uveitis Compared With Patients Without Uveitis, by Duration of Follow-Up^a^

Cataract type	Patients who developed cataract, No. (%) (n = 22 687)		Patients who did not develop cataract, % (n = 22 687)^b^		HR (95% CI)^c^
	With uveitis	Without uveitis	With uveitis	Without uveitis	
**Total duration (up to 20 y)**					
Cataract	803 (3.5)	50 (0.2)	91.65	99.47	17.17 (12.90-22.80)
Infantile, juvenile, and presenile	168 (0.7)	10 (<0.1)	98.02	99.91	17.80 (9.40-33.68)
Traumatic	158 (0.7)	10 (<0.1)	99.06	99.94	27.10 (11.99-61.20)
Complicated	210 (0.9)	10 (<0.1)	97.72	99.90	111.29 (27.65-447.00)
Drug induced	37 (0.2)	10 (<0.1)	99.51	99.96	7.82 (3.08-19.90)
After (secondary)	176 (0.8)	10 (<0.1)	97.65	99.93	23.29 (11.46-47.30)
Unspecified	451 (2.0)	39 (0.2)	94.62	99.56	12.31 (8.88-17.07)
**1-y Follow-up**					
Cataract	402 (1.8)	15 (0.1)	97.83	99.92	27.87 (16.64-46.60)
Infantile, juvenile, and presenile	71 (0.3)	10 (<0.1)	99.60	99.99	37.04 (9.09-150.90)
Traumatic	132 (0.6)	10 (<0.1)	99.32	99.99	67.31 (16.65-271.00)
Complicated	78 (0.3)	0	99.57	100.00	NA
Drug induced	10 (<0.1)	10 (<0.1)	99.95	99.99	9.41 (1.19-74.24)
After (secondary)	51 (0.2)	10 (<0.1)	99.72	99.99	26.56 (6.47-109.00)
Unspecified	165 (0.7)	11 (<0.1)	99.10	99.94	15.57 (8.46-28.67)
**2-y Follow-up**					
Cataract	541 (2.4)	27 (0.1)	96.90	99.85	21.09 (14.32-31.00)
Infantile, juvenile, and presenile	102 (0.4)	10 (<0.1)	99.39	99.98	35.72 (11.33-112.00)
Traumatic	143 (0.6)	10 (<0.1)	99.24	99.98	48.80 (15.55-153.00)
Complicated	120 (0.5)	0	99.29	100.00	NA
Drug induced	12 (0.1)	10 (<0.1)	99.93	99.99	6.30 (1.41-28.14)
After (secondary)	83 (0.4)	10 (<0.1)	99.50	99.99	43.59 (10.72-177.00)
Unspecified	254 (1.1)	22 (0.1)	98.50	99.87	12.13 (7.85-18.75)
**5-y Follow-up**					
Cataract	690 (3.0)	41 (0.2)	95.49	99.72	17.93 (13.08-24.50)
Infantile, juvenile, and presenile	139 (0.6)	10 (<0.1)	99.04	99.93	16.38 (8.35-32.13)
Traumatic	153 (0.7)	10 (<0.1)	99.15	99.97	39.31 (14.56-106.00)
Complicated	171 (0.8)	10 (<0.1)	98.80	99.99	181.48 (25.41-1295.00)
Drug induced	28 (0.1)	10 (<0.1)	99.78	99.97	7.46 (2.62-21.25)
After (secondary)	131 (0.6)	10 (<0.1)	99.04	99.95	19.90 (9.30-42.55)
Unspecified	367 (1.6)	32 (0.1)	97.42	99.78	12.20 (8.50-17.51)

Abbreviations: HR, hazard ratio; NA, not applicable.

^a^ If the patient’s count is 1 to 10, the results indicate a number (percentage) of 10 (<0.1).

^b^ Because of the TriNetX platform’s limitations, raw number (percentage) of patients cannot be included.

^c^ The HR was calculated for each study stratum to determine the risk of developing cataracts among those with uveitis compared with those without uveitis.

At the 1-year time point following the index date, patients younger than 18 years with uveitis were associated with an overall increased risk of developing cataracts (HR, 27.87; 95% CI, 16.64-46.60). This increased risk was evident across various categories of cataracts, including infantile, juvenile, and presenile cataract (HR, 37.04; 95% CI, 9.09-150.90); traumatic cataract (HR, 67.31; 95% CI, 16.65-271.00); drug-induced cataract (HR, 9.41; 95% CI, 1.19-74.24); after cataract (HR, 26.56; 95% CI, 6.47-109.00); and unspecified cataract (HR, 15.57; 95% CI, 8.46-28.67).

At the 2-year time point following the index date, patients younger than 18 years with uveitis an overall increased risk of developing cataracts (HR, 21.09; 95% CI, 14.32-31.00). This increased risk was evident across various categories of cataracts, including infantile, juvenile, and presenile cataract (HR, 35.72; 95% CI, 11.33-112.00); traumatic cataract (HR, 48.80; 95%CI, 15.55-153.00); drug-induced cataract (HR, 6.30; 95%CI, 1.41-28.14); after cataract (HR, 43.59; 95% CI, 10.72-177.00); and unspecified cataract (HR, 12.13; 95% CI, 7.85-18.75).

At the 5-year time point following the index date, patients younger than 18 years with uveitis had an overall increased risk of developing cataracts (HR, 17.93; 95% CI, 13.08-24.50). This increased risk was evident across various categories of cataracts, including infantile, juvenile, and presenile cataract (HR, 16.38; 95% CI, 8.35-32.13); traumatic cataract (HR, 39.31; 95% CI,14.56-106.00); complicated cataract (HR, 181.48; 95% CI, 25.41-1295.00); drug-induced cataract (HR, 7.46; 95% CI, 2.62-21.25); after cataract (HR, 19.90; 95% CI, 9.30-42.55); and unspecified cataract (HR, 12.20; 95% CI, 8.50-17.51).

At the 20-year time point following the index date, patients younger than 18 years with uveitis had an overall increased risk of developing cataracts (HR, 17.17; 95% CI, 12.90-22.80). This increased risk was evident across various categories of cataracts, including infantile, juvenile, and presenile cataract (HR, 17.80; 95% CI, 9.40-33.68); traumatic cataract (HR, 27.10; 95% CI, 11.99-61.20); complicated cataract (HR, 111.29; 95% CI, 27.65-447.00); drug-induced cataract (HR, 7.82; 95% CI, 3.08-19.90); after cataract (HR, 23.29; 95% CI, 11.46-47.30); and unspecified cataract (HR, 12.31; 95% CI, 18.88-17.07).

The risk of cataract occurrence within a specific time point after the index diagnosis of uveitis was also analyzed. Increased risk of cataracts was observed at the time points of 1 month (HR, 15.74; 95% CI, 11.59-21.30), 3 months (HR, 13.22; 95% CI, 9.83-17.80]), and 6 months (HR, 12.93; 95% CI, 9.47-17.64) after the index date (eTable 3 in Supplement 1).

Our study also analyzed the risk of cataracts among patients younger than 18 years with uveitis based on the different inclusion periods (eTable 4 in Supplement 1). Among pediatric patients with uveitis from the inclusion period of 2017 to 2022, an increased overall risk was seen for cataract development (HR, 15.96; 95% CI, 11.02-23.10) compared with those without uveitis. Increased risk was also seen within this inclusion period for the *ICD-10-CM* codes associated with infantile, juvenile, and presenile cataract (HR, 19.02; 95% CI, 7.73-46.84); traumatic cataract (HR, 15.40; 95% CI, 6.23-38.07); complicated cataract (HR, 63.89; 95% CI, 15.78-258.00); after cataract (HR, 24.52; 95% CI, 9.00-66.78); and unspecified cataract (HR, 14.22; 95% CI, 8.92-22.67) among patients younger than 18 years with uveitis compared with those without uveitis. Among pediatric patients with uveitis from the inclusion period of 2011 to 2016, an increased overall risk was seen for cataract development (HR, 11.98; 95% CI, 8.48-16.94) compared with those without uveitis. Increased risk was also seen within this inclusion period for the *ICD-10-CM* codes associated with infantile, juvenile, and presenile cataract (HR, 14.91; 95% CI, 6.51-34.17); traumatic cataract (HR, 6.23; 95% CI, 3.19-12.18); drug-induced cataract (HR, 24.46; 95% CI, 3.30-181.40); after cataract (HR, 10.29; 95% CI, 5.36-19.75); and unspecified cataracts (HR, 15.28; 95% CI, 9.34-25.00) among patients younger than 18 years with uveitis compared with those without uveitis.

Our study also assessed the risk of cataract development within a specific time frame after the index date based on the *ICD-10-CM* codes corresponding to the anatomical location of the uveitis (eTable 5 in Supplement 1). Patients younger than 18 years with anterior uveitis had an increased risk of cataract formation at the 1-month (HR, 15.07; 95% CI, 7.95-28.59), 2-month (HR, 17.60; 95% CI, 9.59-32.32), 3-month (HR, 16.36; 95% CI, 9.34-28.65), 6-month (HR, 20.15; 95% CI, 11.53-35.10), 9-month (HR, 21.50; 95% CI, 12.80-36.10), 12-month (HR, 23.83; 95% CI, 14.20-39.90), 18-month (HR, 22.56; 95% CI, 14.24-35.70), and 24-month (HR, 21.18; 95% CI, 13.93-32.10) time point after the index date compared with those without uveitis. Patients younger than 18 years with intermediate uveitis had an increased risk of cataract formation at the 1-month (HR, 9.84; 95% CI, 1.26-76.87), 2-month (HR, 12.84; 95% CI, 1.68-98.15), 3-month (HR, 11.50; 95% CI, 2.71-48.79), 6-month (HR, 7.58; 95% CI, 2.67-21.50), 9-month (HR, 10.23; 95% CI, 3.66-28.59), 12-month (HR, 11.85; 95% CI, 4.27-32.92), 18-month (HR, 13.47; 95% CI, 4.87-37.25), and 24-month (HR, 11.59; 95% CI, 5.03-26.74) time point after the index date compared with those without uveitis. Among the patients younger than 18 years with posterior uveitis, no increased risk of cataract formation was seen across the different follow-up periods after the index date compared with those without uveitis. Among patients younger than 18 years with panuveitis, no increased risk of cataract formation was seen from the 1-month time point to the 24-month time point after the index date compared with those without uveitis due to the lack of data.

Our study also assessed the risk of cataract development based on the anatomical location and the *ICD-10-CM* codes corresponding to the etiology of the uveitis across the entire study duration among patients younger than 18 years with uveitis compared with those without uveitis (eTable 6 in Supplement 1). In terms of the anatomical location of uveitis, those with anterior uveitis, intermediate uveitis, posterior uveitis, and panuveitis were all found to have increased risk of cataract across the entire study duration compared with those without uveitis (anterior uveitis: HR, 17.37 [95% CI, 12.71-23.70]; intermediate uveitis: HR, 13.07 [95% CI, 6.62-25.78], posterior uveitis: HR, 9.44 [95% CI, 1.18-75.58]; and panuveitis: HR, 42.16 [95% CI, 5.77-308.20]). In terms of the etiology of the respective uveitis, not enough patient data were available to analyze the risk of cataracts among pediatric patients with purulent endophthalmitis, sympathetic uveitis, ophthalmia nodosa, or secondary uveitis infection compared with those without uveitis. The corresponding Kaplan-Meier curves are depicted in Figure 2. When stratified by follow-up duration, a significant association between cataract development and index uveitis diagnosis was seen at the 1-, 2-, 5-, and 20-year follow-up points.

### Subgroup Analysis

The risk of cataracts was further investigated through a subgroup analysis based on sex, age, race and ethnicity, and specific medical comorbidities (eTable 7 and eFigure 2 in Supplement 1). In terms of the age groups assessed, an increased risk of cataracts was seen among patients with uveitis who were aged 0 to 6 years (HR, 19.09; 95% CI, 10.10-36.00), 7 to 12 years (HR, 27.16; 95% CI, 15.59-47.20), and 13 to 18 years (HR, 13.39; 95% CI, 8.84-20.30).

In terms of sex, an increased risk of cataracts was seen among patients with uveitis who were female (HR, 13.76; 95% CI, 9.60-19.71) and male (HR, 11.97; 95% CI, 8.47-16.91). In terms of race and ethnicity, an increased risk of cataracts was seen among patients who were Asian (HR, 13.80; 95% CI, 3.28-58.07), Black or African American (HR, 10.41; 95% CI, 5.60-19.36), and White (HR, 15.82; 95% CI, 11.05-22.60). In terms of comorbidities, an increased risk of cataracts was seen among patients with uveitis who had asthma (HR, 6.65; 95% CI, 2.32-19.04) and juvenile arthritis (HR, 59.31; 95% CI, 8.23-427.60).

### Risk Stratification Analysis

Among patients younger than 18 years with intraocular surgery history within 2 years of the index date, an increased cataract risk was seen among those with (HR, 11.07; 95% CI, 4.42-27.71) compared with those without (HR, 14.09; 95% CI, 10.11-20.70) uveitis disease (eTable 8 and eFigure 3 in Supplement 1). Compared with patients without uveitis, cataract development was associated with those with a history of steroid eye drop use within 3 months after the index date (HR, 29.51; 95% CI, 14.56-59.70). This increased cataract risk associated with steroid eye drop use persisted among patients with uveitis even after a washout period of 3 months (HR, 45.27; 95% CI, 14.38-142.00) (eTable 8 in Supplement 1). Among patients with uveitis who never used steroid eyes drops, an association with cataract development was also seen among patients with uveitis (HR, 16.49; 95% CI, 11.92-22.70) compared with those without uveitis (eTable 9 in Supplement 1). In terms of the risk of cataracts among the different eye drops, increased cataract risk was seen among those receiving prednisolone eye drops (HR, 26.42; 95% CI, 12.39-56.20) and dexamethasone eye drops (HR, 13.34; 95% CI, 1.74-102.60) with uveitis compared with those without uveitis (eTables 8-10 and eFigure 3 in Supplement 1).

Compared with patients without uveitis disease, an association with cataract risk was seen among those with a history of immunosuppressive agents within 3 months after the index date (HR, 26.52; 95% CI, 16.75-41.90). This increased cataract risk persisted even after a washout period of 3 months (HR, 24.35; 95% CI, 14.23-41.60). Elevated HRs associated with cataract risk were also seen among patients with uveitis who received steroidal immunosuppressive agents within 3 months after the index date (HR, 5.62; 95% CI, 4.38-7.20) and those without a history of receiving steroidal immunosuppressive agents within 3 months after the index date (HR, 20.12; 95% CI, 6.31-64.17) compared with those without uveitis. Regarding specific immunosuppressive agents, increased cataract risk was also seen among patients with uveitis who were recipients of prednisolone (HR, 17.97; 95% CI, 9.76-33.08), methylprednisolone (HR, 8.19; 95% CI, 1.00-66.94), triamcinolone (HR, 13.67; 95% CI, 1.79-104.50), dexamethasone (HR, 7.28; 95% CI, 2.16-24.51), and methotrexate (HR, 20.52; 95% CI, 6.42-65.62). Furthermore, an association with cataract risk was also seen among patients with uveitis who did not have a history of immunosuppressive agents (HR, 17.69; 95% CI, 11.39-27.40) compared with patients without uveitis (eTables 8-10 and eFigure 3 in Supplement 1). The use of immunosuppressive agents was defined by RxNorm codes and included prednisolone, betamethasone, methylprednisolone, triamcinolone, dexamethasone, sulfasalazine, azathioprine, methotrexate, cyclosporine, cyclophosphamide, tacrolimus, and leflunomide.

We also conducted subgroup analyses based on inflammatory-related laboratory results, specifically on CRP levels and leukocyte counts. We set a cutoff point of 1 mg/dL for CRP level and 11 000/μL for leukocyte count, as these values are commonly used to indicate elevated inflammation (eTable 9 in Supplement 1).

Among patients younger than 18 years with uveitis, those with CRP levels less than 1 mg/dL had a higher risk of cataract development (HR, 6.06; 95% CI, 2.35-15.62) compared with those without uveitis. Similarly, patients younger than 18 years with uveitis and CRP levels 1 mg/dL or more also had an increased risk of cataract development (HR, 13.80; 95% CI, 4.26-44.65) compared with those without uveitis.

Regarding leukocyte counts, patients younger than 18 years with uveitis and leukocyte counts less than 11 000/μL had higher risk of cataract development (HR, 9.41; 95% CI, 5.18-17.09) compared with those without uveitis. Likewise, among patients younger than 18 years with uveitis and leukocyte counts 11 000/μL or more also had a higher risk of cataract development (HR, 9.64; 95% CI, 3.44-27.04) compared with those without uveitis. Furthermore, among patients with uveitis, our subgroup analysis still revealed an increased cataract risk among patients with uveitis even after the exclusion of trauma-related cataracts (HR, 14.81; 95% CI, 11.01-19.90) compared with the reference nonuveitis group (eTable 8 in Supplement 1).

We also conducted a risk-stratification analysis based on a previous history of autoimmune diseases (eTable 10 in Supplement 1). Our findings revealed that patients with uveitis with a history of autoimmune disease (HR, 11.51; 95% CI, 5.32-24.92) had elevated HRs associated with cataract formation compared with their respective comparators without uveitis. Patients with uveitis who did not have a history of autoimmune disease (HR, 12.94; 95% CI, 9.96-16.79) also had elevated HRs associated with cataract development compared with their respective comparators without uveitis.

### Positive and Negative Controls

Our study also explored the risk of being prescribed steroid eye drops among patients younger than 18 years with uveitis compared with those without uveitis as positive outcome controls. Given the widespread acceptance of steroid eye drops in the management of uveitis, this investigation aimed to assess if our current study design could reaffirm widely accepted associations. Additionally, we delved into the risk associated with osteoporosis among patients younger than 18 years with uveitis as negative outcome controls. Since such associations were not expected to demonstrate an association among pediatric patients with uveitis, such an analysis also served to support our study design’s robustness (eTable 11 in Supplement 1). As part of our positive outcome control, the risk of steroid eye drop use among pediatric patients with uveitis was found to be elevated compared with those without uveitis (HR, 3.73; 9.5% CI, 3.36-4.14). As part of our negative outcome control, there were no associations among patients younger than 18 years with uveitis and the risk of osteoporosis (HR, 1.50; 95% CI, 0.88-2.56).

### Additional Analysis Based on the EMEA TriNetX Network

The main analysis performed for this study was based on the US Collaborative Network in TriNetX. We also examined the risk of incident cataracts among patients younger than 18 years with uveitis from the EMEA regional network using the same study design from our previous analysis conducted in the US collaborative network.

In total, 1791 patients younger than 18 years with uveitis (mean [SD] age at index, 9.7 [5.3]; 48.1% female; 51.9% male) and 1791 patients younger than 18 years without uveitis (mean [SD] age at index, 9.7 [5.3]; 47.7% female; 52.3% male) were enrolled from the EMEA TriNetX network (eTable 12 in Supplement 1). The 2 cohorts were propensity-matched with a 1:1 ratio based on identical covariates mentioned previously. In terms of risk for cataracts among patients younger than 18 years with uveitis after 20 years of follow-up, an increased overall risk for cataracts was seen (HR, 7.23; 95% CI, 2.81-18.59) compared with those without uveitis. Furthermore, increased cataract risk was seen for the *ICD-10-CM* codes corresponding to complicated cataract (HR, 4.80; 95% CI, 1.36-16.88) and unspecified cataract (HR, 8.57; 95% CI, 1.94-37.8) (eTable 13 in Supplement 1).

### External Validation Analysis

Our findings underwent further validation using datasets from the Taiwan NHIRD. Using a similar study design, we enrolled 1129 pediatric patients with uveitis and 11 290 propensity score–matched pediatric patients without uveitis. A 1:10 propensity score–matching ratio to pair patients younger than 18 years with uveitis with those without uveitis was chosen for this external validation analysis due to the limited patient numbers available in the NHIRD. Among this enrolled population, the findings showed increased cataract risk among patients with uveitis compared with patients without uveitis (adjusted HR, 17.2; 95% CI, 13.89-21.30; *P* < .001) (eTable 14 and eFigure 4 in Supplement 1).

## Discussion

In this cohort study, our findings showed an increased risk of cataract development among pediatric patients younger than 18 years with uveitis compared with those without. The risk for cataracts persisted throughout all follow-up durations, extending up to 20 years from the index date. The association with uveitis was observed even after trauma-related and age-related cataracts were excluded.

Furthermore, subgroup analyses revealed an increased cataract risk among pediatric patients with uveitis across the ages of 0 to 18 years among both sexes; patients who were Asian, Black or African American, or White; and patients with medical comorbidities of asthma and juvenile arthritis. Our study also noted an association between cataract risk in pediatric patients with uveitis among those with and without a history of immunosuppressive agents or steroid eye drop use. Although cataract risk was associated with patients younger than 18 years with uveitis who had both elevated and nonelevated levels of CRP and leukocyte count, we also observed a higher HR value associated with cataract risk among pediatric patients with uveitis who had elevated levels of CRP and leukocyte count. Conversely, we noted a lower magnitude of HR associated with cataract risk among pediatric patients with uveitis who had lower levels of CRP and leukocyte count.

Our study has relevant clinical implications. The study provides valuable insights into the incidence of cataracts following uveitis diagnosis among pediatric patients with uveitis. It is notable for being one of the largest studies to date in this field and for using rigorous methods, including propensity score matching, extended follow-up periods, the inclusion of positive and negative outcome controls, and the performance of 2 different regional analyses within the TriNetX network. Our study results have also been externally validated with the Taiwan NHIRD, reinforcing our findings’ validity. Our findings are of importance, as cataracts can significantly impact vision and may lead to eventual blindness if not promptly diagnosed and treated.

We summarized relevant studies in the literature^2,9,10,11,15,16,17,18^ concerning cataract risk among patients with uveitis in eTable 15 in Supplement 1. In a retrospective study by Minkus et al,^2^ the authors recruited 2190 patients with intermediate uveitis to assess the risk of cataract formation. Their findings showed an increased risk of cataracts among patients with uveitis, with a cumulative incidence rate of 36.6% by 10 years. These results were partially complementary to our own. Our study revealed that patients younger than 18 years diagnosed with anterior and intermediate uveitis exhibited a heightened risk of developing cataracts throughout the follow-up period ranging from 1 month to 24 months after the index date compared with those without uveitis (eTable 5 in Supplement 1). One point to note from the study by Minkus et al^2^ is that their findings did not show any association between race and ethnicity and sex with the risk of cataract development. This contrasts with our study, in which we found increased risk among both sexes and patients who were Asian, Black or African American, or White. Our supplementary analysis, using the Taiwan NHIRD that was based predominantly on Asian patients, also supported the heightened risk of cataracts among patients younger than 18 years with uveitis compared with those without uveitis. A brief literature review also found an increased risk of cataracts among females and males due to sex-related hormones.^19,20^ There were also reports of increased cataract risk among Black or African American and White patients.^20,21^ It is unknown why Minkus et al^2^ found no association concerning sex or race and ethnicity, but perhaps the age of the patients recruited may have influenced such associations. Minkus et al^2^ did not restrict the age of recruited patients. In contrast, our study enrolled only patients 18 years or younger. Younger patients have reported experiencing more severe forms of intraocular formation than their older counterparts. A study by Lai et al^6^ hypothesized that this might be related to the immature blood-ocular barrier present among younger patients, as the blood-ocular barrier plays a role in maintaining the immune-privileged intraocular environment. Such immaturity in intraocular structures may explain these findings.

Another noteworthy point from Minkus et al^2^ was that uveitis eyes with concurrent anterior and posterior synechiae, an epiretinal membrane, and a high dose of corticosteroid therapy were found to be at an increased risk of cataract development. Similar findings were reported elsewhere.^16,17^ All of these factors may indicate the baseline severity of uveitis. It stands to reason that more severe uveitis at the onset would heighten the risk of cataracts at the final follow-up. Although our study did not specifically assess the presence of synechiae, epiretinal membranes, or doses of corticosteroid therapy due to limitations inherent in diagnostic codes, we did investigate the association of specific laboratory findings of inflammation with the risk of developing cataracts. First, our study found that the baseline CRP and leukocyte levels were higher among patients younger than 18 years with uveitis than among those without uveitis (Table 2). This hints toward the elevated baseline inflammatory state among pediatric patients with uveitis. Second, we also conducted a subgroup analysis based on laboratory results commonly associated with inflammation. Our analysis revealed an elevated risk of cataract development among patients with uveitis, particularly among those possessing CRP levels less than 1 mg/dL and 1 mg/dL or more. The HR for cataract risk was notably higher for those with CRP levels 1 mg/dL or more compared with those without uveitis. Similarly, we found that patients with uveitis possessing leukocyte counts less than 11 000/μL had an elevated risk of cataracts, as did those with leukocyte counts of 11 000/μL or more. The HR for cataract risk was higher among those with leukocyte counts of 11 000/μL or more compared with those without uveitis. These combined findings support the potential pathophysiological links based on inflammation between uveitis and the subsequent development of cataracts. These findings also indirectly support the conclusion by Minkus et al^2^ that the initial severity of uveitis may be associated with an elevated risk of cataract development.

As part of our subgroup analysis, we observed an increased risk of specific *ICD-10-CM*–defined subtypes of cataract (specifically, infantile, juvenile, and presenile; traumatic; complicated; drug induced; and unspecified) compared with the nonuveitis group for up to 20 years after the index uveitis date. Using *ICD-10-CM* codes for defining cataracts introduces challenges in generalizability to other studies, as not all studies rely on *ICD-10-CM* codes.^10^ Our study may be the first to date to assess cataract risk after uveitis diagnosis based on diagnostic codes from an electronic health claims database.

In terms of steroid therapy, our results also found increased cataract risk among those who had a history of steroid eye drop use. However, Thorne et al^18^ noted that steroid eye drops dosed at 3 drops or less daily seemed to be associated with a lower risk of cataract development among pediatric uveitis eyes than those receiving a higher dose. It should be highlighted that receiving more steroid eye drops may again reflect the severity of the uveitis. In other words, those with higher doses of steroid eye drops from the study by Thorne et al^18^ may also involve patients with more severe uveitis. More severe uveitis may result in an increased risk of cataracts seen among the patients of Thorne et al.^18^ While the TriNetX platform limits analysis regarding the frequency of steroid eye drop dosage, our study conducted subgroup analyses based on individual steroid eye drop use. We assessed the risk of cataracts among patients with uveitis who were recipients of prednisolone and dexamethasone eye drops, as well as those who did not use any steroid eye drops. Our findings revealed that patients with uveitis who received prednisolone and dexamethasone eye drops were all associated with cataract development compared with those without uveitis. Additionally, we observed a lower magnitude of cataract development among patients with uveitis who did not receive steroid eye drops (HR, 16.49; 95% CI, 11.92-22.70) compared with those who did (HR, 29.51; 95% CI, 14.56-59.70) when compared with individuals without uveitis. Our findings, therefore, support the observations of Thorne et al,^18^ indicating that patients with uveitis who received steroid eye drops exhibited a higher HR associated with cataracts than those who did not receive such treatment. However, it’s important to note that our study cannot rule out the possibility that individuals receiving steroid eye drops may inherently have more severe cases of uveitis. Future studies are still needed to confirm our findings further.

Furthermore, our findings showed a lower HR for cataract development among individuals younger than 18 years with a history of intraocular surgeries than among those without (eTable 8 in Supplement 1). Considering the anticipated inflammation caused by intraocular procedures, a possible reason could be the confounding effects of medications. Specifically, certain anti-inflammatories, like topical nonsteroidal anti-inflammatories, are frequently administered to patients undergoing specific intraocular procedures.^22^ Such adjuvant drugs might have mitigated inflammation within the intraocular structures caused by the procedures, potentially leading to a reduced risk of cataract development.^23^

Our results further revealed that using immunosuppressive agents among patients with uveitis was associated with an increased cataract risk. Notably, this association persisted even after a 3-month washout period. Findings of a separate risk-stratification analysis conducted by our study showed HRs of a lower magnitude for steroidal immunosuppressive agents (HR, 5.62; 95% CI, 4.38-7.20) compared with nonusers of steroidal immunosuppressive agents (HR, 20.12; 95% CI, 6.31-64.17) (eTable 10 in Supplement 1). It is possible that the use of these immunosuppressive agents might potentially mitigate inflammatory pathways linked to uveitis, thereby reducing the likelihood of developing cataracts. However, despite these findings, we also found that methotrexate showed the highest magnitude in terms of HRs for cataract development among recipients with uveitis, followed by prednisolone and triamcinolone (eTable 9 in Supplement 1). These findings hold clinical relevance, as they suggest that clinicians should still exercise caution when prescribing such medications to pediatric patients with uveitis due to the associated risk of cataract development. Furthermore, as we lacked information on the effects of dosage, duration of therapy, and route of administration, future studies are still necessary to address any unanswered questions.

In addition, we also assessed whether the diagnosis of autoimmune diseases may have been associated with the risk of cataract formation. Our results suggest a greater magnitude of HRs associated with cataracts among those without autoimmune disease compared with those with autoimmune disease. Inflammation is a key underlying pathophysiology contributing to autoimmune disease and cataracts.^24^ Findings from a previous study have shown disease duration and severity of systemic lupus erythematosus to be significantly associated with cataract formation,^25^ and we believe such factors may have influenced our results.

### Limitations

This study has limitations. First, as our data relied on the retrospective analysis of health care-related diagnostic codes, there were certain factors that we could not analyze. For instance, we could not assess the severity of the cataract in question. Another issue was the lack of information on the dosage and frequency of the steroid eye drops and the immunosuppressive medications used. We also could not evaluate the potential impact of specific procataract genetic mutations on the risk of developing cataracts following a diagnosis for uveitis. Another issue pertains to the regional coverage of the TriNetX platform. The TriNetX platform is organized into various networks based on regions, with the US network being the primary focus of our analysis due to its substantial patient volume compared with the other networks. According to the October 2022 data, the US network accounted for approximately 134 million patients out of the global 171 million patients within the TriNetX platform. In contrast, the EMEA network, covering Europe, the Middle East, and Africa, comprised only 32 million patients.^26^ Our study population from the US network comprised 2.8% Asian and 19.5% Black or African American individuals without uveitis younger than 18 years, thereby restricting the generalizability of our findings to other races and ethnicities. In an attempt to address this limitation, we conducted separate analyses using the EMEA network of TriNetX and the Taiwan NHIRD. Our analysis using the Taiwan NHIRD focused on a study population predominantly composed of Asian individuals, supporting the increased risk of cataracts among Asian patients younger than 18 years with uveitis compared with those without uveitis. However, in terms of our analysis based on the EMEA network, while it also supported the increased risk of cataracts among patients with uveitis compared with those without using a distinct regional EMEA network of TriNetX, most of the EMEA study population data that we obtained had unspecified race and ethnicity, with only 0.6% categorized as Black or African American and 4.3% as White. This limited representation of racial and ethnic diversity in the EMEA network hinders any associations regarding the risk of cataracts in patients with uveitis among Black or African American individuals or other racial and ethnic minority groups. Future studies involving local databases from African hospitals may be warranted to address any remaining unanswered questions regarding this topic. The issue of medication nonadherence is also relevant, especially considering our study’s focus on pediatric patients. Medication nonadherence among patients younger than 18 years has been widely recognized as a significant challenge, with an estimated average nonadherence rate of approximately 50% reported in 1 study.^27^ These behavioral factors can potentially impact our findings, since medication adherence has been shown to affect the clinical course of inflammatory eye diseases.^28^ Nonadherence to medications could exacerbate and prolong the clinical course of inflammatory eye diseases, potentially leading to an overestimation of cataract risks within our patient cohort secondary to overactivation of the inflammatory pathways. Such effects, secondary to nonadherence to medication, were hinted at within our subgroup analysis, in which the findings showed a higher HR for cataract development among patients with uveitis who did not receive steroidal immunosuppressive agents compared with those who did (eTable 10 in Supplement 1). However, our retrospective study design can suggest potential associations but cannot definitively establish causation. Future prospective studies are required to definitively determine the effects of medication nonadherence on cataract risk among patients younger than 18 years with uveitis.

Another limitation of our study design pertains to residual or unmeasured confounding factors that could have influenced the study results. These confounders, which could have been more effectively controlled through matching based on follow-up duration and index date, were not addressed in this manner by our study due to limitations inherent to the TriNetX platform. To mitigate this limitation, we analyzed cataract risks based on different inclusion periods for uveitis, specifically between 2017 and 2022 and between 2011 and 2016 (eTable 4 in Supplement 1). Through this analysis, we still observed elevated HRs associated with cataract risk among patients younger than 18 years with uveitis between these 2 inclusion periods compared with those without uveitis, which supports our study design. However, it is important to note that such an analysis based on inclusion periods may not completely account for all residual confounding factors, which remains a limitation to be acknowledged.

A final limitation of our study pertains to the limited information available regarding the effects of inflammation mediators on the overall risk of cataracts among patients younger than 18 years with uveitis. These mediators include proinflammatory cytokines, genetic profiles, and structural factors such as the immaturity of the blood-ocular barrier (eFigure 5 in Supplement 1). Limited data availability hindered our attempts to analyze genetic profiles within TriNetX. It was also impossible to evaluate for structural profile information on the blood-ocular barriers of our enrolled patients from the TriNetX data. However, within the TriNetX database, we could assess for certain proinflammatory markers using laboratory results. Specifically, we focused on 2 commonly used markers to assess patients’ systemic inflammatory states: CRP and leukocyte levels. Our findings revealed higher baseline mean CRP leukocyte levels among patients with uveitis than among those without uveitis. Furthermore, findings from our subgroup analysis based on these inflammatory markers showed higher HRs associated with cataract risks among patients with uveitis with CRP levels 1 mg/dL or more compared with those with CRP levels less than 1 mg/dL. Similarly, we observed higher HRs associated with cataract risks among patients with uveitis with leukocyte counts of 11 000/μL or more compared with those with leukocyte counts less than 11 000/μL. Although our results suggest the association of certain systemic inflammatory mediators with the risk of cataract formation among patients with uveitis, we are limited in our ability to draw further conclusions regarding the associations of other inflammatory cytokine levels, such as intraocular levels of inflammatory cells, genetic profiles related to cataracts, and the structural maturity of the blood-ocular barrier with cataract risk.

## Conclusions

This cohort study found that pediatric patients younger than 18 years with uveitis diseases may be at a significantly increased risk for cataract development. These findings suggest that pediatric patients with uveitis should be monitored for future cataract development.

## Notes

Supplement 1. **eTable 1.** Code for Intraocular Surgery **eTable 2.** Code for Medications **eTable 3.** Risk of Cataract Exposure in Uveitis Versus Nonuveitis Groups Across Different Inclusion Periods **eTable 4.** Risk of Cataract Exposure by Follow-Up Duration of Different Aetiology of Uveitis and Anatomical Location **eTable 5.** Risk of Cataract Exposure by Different Aetiology of Uveitis and Anatomical Location Uveitis **eTable 6.** Risk of Cataract Exposure by Different Aetiology of Uveitis and Anatomical Location Uveitis **eTable 7.** Stratification for Risk of Uveitis Exposed to Uveitis Compared With Nonuveitis **eTable 8.** Sensitivity Analysis for Risk of Cataract Exposed to Uveitis Compared With Nonuveitis **eTable 9.** Risk of Cataract Exposed to Uveitis Compared With Nonuveitis by Steroid Eye Drop, Immunosuppressive Agents, C-Reactive Protein, and Leukocytes **eTable 10.** Stratification Analysis for Risk of Cataract Exposed to Uveitis Compared With Nonuveitis **eTable 11.** Positive Outcome Control, Negative Outcome Control Analysis **eTable 12.** Demographic Characteristics of Uveitis and Nonuveitis by Using the EMEA Network **eTable 13.** Risk of Cataract Exposed to Uveitis Compared With Nonuveitis by Using the EMEA Network **eTable 14.** Validation Analysis Using the Taiwan Insurance Research Database on the Risk of Cataract Among Patients With Uveitis **eTable 15.** Summary of Studies **eFigure 1.** Brief Overview of the Study Design **eFigure 2.** Forest Plot of Stratification Analysis for Risk of Cataract **eFigure 3.** Forest Plot of Sensitivity Analysis for Risk of Cataract Exposed to Uveitis Compared With Nonuveitis **eFigure 4.** Kaplan-Meier Curves for Risk of Cataract Among Patients With Uveitis From the Taiwan Health Insurance Database **eFigure 5.** Proposed Pathophysiology Contributing to Cataract Formation Among Pediatric Uveitis Eyes

Supplement 2.
Data Sharing Statement
