# Supplementary material for: Cataract Development Among Pediatric Patients With Uveitis
Source: JAMA Netw Open. 2024 Jul 1;7(7):e2419366. doi: 10.1001/jamanetworkopen.2024.19366 (PMC11217876; doi:10.1001/jamanetworkopen.2024.19366)
Supplement: Supplement 1. — eTable 1. Code for Intraocular Surgery eTable 2. Code for Medications eTable 3. Risk of Cataract Exposure in Uveitis Versus Nonuveitis Groups Across Different Inclusion Periods eTable 4. Risk of Cataract Exposure by Follow-Up Duration of Different Aetiology of Uveitis and Anatomical Location eTable 5. Risk of Cataract Exposure by Different Aetiology of Uveitis and Anatomical Location Uveitis eTable 6. Risk of Cataract Exposure by Different Aetiology of Uveitis and Anatomical Location Uveitis eTable 7. Stratification for Risk of Uveitis Exposed to Uveitis Compared With Nonuveitis eTable 8. Sensitivity Analysis for Risk of Cataract Exposed to Uveitis Compared With Nonuveitis eTable 9. Risk of Cataract Exposed to Uveitis Compared With Nonuveitis by Steroid Eye Drop, Immunosuppressive Agents, C-Reactive Protein, and Leukocytes eTable 10. Stratification Analysis for Risk of Cataract Exposed to Uveitis Compared With Nonuveitis eTable 11. Positive Outcome Control, Negative Outcome Control Analysis eTable 12. Demographic Characteristics of Uveitis and Nonuveitis by Using the EMEA Network eTable 13. Risk of Cataract Exposed to Uveitis Compared With Nonuveitis by Using the EMEA Network eTable 14. Validation Analysis Using the Taiwan Insurance Research Database on the Risk of Cataract Among Patients With Uveitis eTable 15. Summary of Studies eFigure 1. Brief Overview of the Study Design eFigure 2. Forest Plot of Stratification Analysis for Risk of Cataract eFigure 3. Forest Plot of Sensitivity Analysis for Risk of Cataract Exposed to Uveitis Compared With Nonuveitis eFigure 4. Kaplan-Meier Curves for Risk of Cataract Among Patients With Uveitis From the Taiwan Health Insurance Database eFigure 5. Proposed Pathophysiology Contributing to Cataract Formation Among Pediatric Uveitis Eyes [file jamanetwopen-e2419366-s001.pdf]

Supplemental Online Content

Hsu AY, Kuo H-T, Lin C-J, et al. Cataract development among pediatric patients with uveitis. *JAMA Netw Open*. 2024;7(7):e2419366.
doi:10.1001/jamanetworkopen.2024.19366

eTable 1. Code for Intraocular Surgery
eTable 2. Code for Medications
eTable 3. Risk of Cataract Exposure in Uveitis Versus Nonuveitis Groups Across Different Inclusion Periods
eTable 4. Risk of Cataract Exposure by Follow-up Duration of Different Aetiology of Uveitis and Anatomical Location
eTable 5. Risk of Cataract Exposure by Different Aetiology of Uveitis and Anatomical Location Uveitis
eTable 6. Risk of Cataract Exposure by Different Aetiology of Uveitis and Anatomical Location Uveitis
eTable 7. Stratification for Risk of Uveitis Exposed to Uveitis Compared With Nonuveitis
eTable 8. Sensitivity Analysis for Risk of Cataract Exposed to Uveitis Compared With Nonuveitis
eTable 9. Risk of Cataract Exposed to Uveitis Compared With Nonuveitis by Steroid Eye Drop, Immunosuppressive Agents, C-Reactive Protein, and Leukocytes
eTable 10. Stratification Analysis for Risk of Cataract Exposed to Uveitis Compared With Nonuveitis
eTable 11. Positive Outcome Control, Negative Outcome Control Analysis
eTable 12. Demographic Characteristics of Uveitis and Nonuveitis by Using the EMEA Network
eTable 13. Risk of Cataract Exposed to Uveitis Compared With Nonuveitis by Using the EMEA Network
eTable 14. Validation Analysis Using the Taiwan Insurance Research Database on the Risk of Cataract Among Patients With Uveitis
eTable 15. Summary of Studies
eFigure 1. Brief Overview of the Study Design
eFigure 2. Forest Plot of Stratification Analysis for Risk of Cataract
eFigure 3. Forest Plot of Sensitivity Analysis for Risk of Cataract Exposed to Uveitis Compared With Nonuveitis
eFigure 4. Kaplan-Meier Curves for Risk of Cataract Among Patients With Uveitis From the Taiwan Health Insurance Database
eFigure 5. Proposed Pathophysiology Contributing to Cataract Formation Among Pediatric Uveitis Eyes

This supplemental material has been provided by the authors to give readers additional information about their work.

© 2024 Hsu AY et al. *JAMA Network Open*.

eTable 1. Code for Intraocular Surgery

Item	Code
Insertion of aqueous drainage device, without extraocular reservoir, internal approach, into the subconjunctival space; initial device	CPT = 0449T
Surgical Procedures on the Anterior Segment of the Eye	CPT = 1009771
Removal of foreign body, intraocular; from anterior chamber of eye or lens	CPT = 65235
Removal of foreign body, intraocular; from posterior segment, magnetic extraction, anterior or posterior route	CPT = 65260
Removal of foreign body, intraocular; from posterior segment, nonmagnetic extraction	CPT = 65265
Repair of laceration; cornea and/or sclera, perforating, not involving uveal tissue	CPT = 65280
Repair of laceration; cornea and/or sclera, perforating, with reposition or resection of uveal tissue	CPT = 65285
Repair of laceration; application of tissue glue, wounds of cornea and/or sclera	CPT = 65286
Removal of corneal epithelium; with or without chemocauterization (abrasion, curettage)	CPT = 65435
Paracentesis of anterior chamber of eye (separate procedure); with removal of aqueous	CPT = 65800
Paracentesis of anterior chamber of eye (separate procedure); with removal of vitreous and/or discission of anterior hyaloid membrane, with or without air injection	CPT = 65810
Paracentesis of anterior chamber of eye (separate procedure); with removal of blood, with or without irrigation and/or air injection	CPT = 65815
Goniotomy	CPT = 65820
Trabeculotomy ab externo	CPT = 65850
Trabeculoplasty by laser surgery	CPT = 65855
Injection, anterior chamber of eye (separate procedure); air or liquid	CPT = 66020
Injection, anterior chamber of eye (separate procedure); medication	CPT = 66030
Fistulization of sclera for glaucoma; trabeculectomy ab externo in absence of previous surgery	CPT = 66170
Fistulization of sclera for glaucoma; trabeculectomy ab externo with scarring from previous ocular surgery or trauma (includes injection of antifibrotic agents)	CPT = 66172
Transluminal dilation of aqueous outflow canal; without retention of device or stent	CPT = 66174
Transluminal dilation of aqueous outflow canal; with retention of device or stent	CPT = 66175
Aqueous shunt to extraocular equatorial plate reservoir, external approach; without graft	CPT = 66179
Aqueous shunt to extraocular equatorial plate reservoir, external approach; with graft	CPT = 66180
Iridectomy, with corneoscleral or corneal section; peripheral for glaucoma (separate procedure)	CPT = 66625
Iridoplasty by photocoagulation (1 or more sessions) (eg, for improvement of vision, for widening of anterior chamber angle)	CPT = 66762
Unlisted procedure, anterior segment of eye	CPT = 66999
Injection of vitreous substitute, pars plana or limbal approach (fluid-gas exchange), with or without aspiration (separate procedure)	CPT = 67025
Intravitreal injection of a pharmacologic agent (separate procedure)	CPT = 67028
Vitrectomy, mechanical, pars plana approach	CPT = 67036
Vitrectomy, mechanical, pars plana approach; with focal endolaser photocoagulation	CPT = 67039
Vitrectomy, mechanical, pars plana approach; with endolaser panretinal photocoagulation	CPT = 67040
Vitrectomy, mechanical, pars plana approach; with removal of preretinal cellular membrane (eg, macular pucker)	CPT = 67041
Vitrectomy, mechanical, pars plana approach; with removal of internal limiting membrane of retina (eg, for repair of macular hole, diabetic macular edema), includes, if performed, intraocular tamponade (ie, air, gas or silicone oil)	CPT = 67042
Vitrectomy, mechanical, pars plana approach; with removal of subretinal membrane (eg, choroidal neovascularization), includes, if performed, intraocular tamponade (ie, air, gas or silicone oil) and laser photocoagulation	CPT = 67043
Repair of retinal detachment; by injection of air or other gas (eg, pneumatic retinopexy)	CPT = 67110
Scleral reinforcement (separate procedure); without graft	CPT = 67250

© 2024 Hsu AY et al. *JAMA Network Open*.

Unlisted procedure, posterior segment	CPT = 67299
Unlisted procedure, conjunctiva	CPT = 68399
Displacement of other ocular prosthetic devices, implants and grafts	ICD-10-CM = T85.328
Corneal transplant status	ICD-10-CM = Z94.7
Other specified postprocedural states	ICD-10-CM = Z98.89

Abbreviations: CPT, Current Procedural Terminology; ICD-10-CM, International Classification of Diseases, Tenth Revision, Clinical Modification.

© 2024 Hsu AY et al. *JAMA Network Open*.

eTable 2. Code for Medications

Item	RxNorm code
Steroid eye drop	
Prednisolone (route: ophthalmic product)	8638
Dexamethasone (route: ophthalmic product)	3264
Hydrocortisone (route: ophthalmic product)	5492
Immunosuppressive agents	
Prednisolone	8638
Dexamethasone	3264
Betamethasone	1514
Triamcinolone	10759
Methylprednisolone	6902
Cyclosporine	3008
Sulfasalazine	9524
Azathioprine	1256
Methotrexate	6851
Cyclophosphamide	3002
Tacrolimus	42316
Leflunomide	27169

Abbreviations: RxNorm, A standardized, controlled terminology for medications in the United States.

eTable 3. Risk of Cataract Exposure in Uveitis Versus Nonuveitis Groups Across Different Inclusion Periods

	Uveitis		Non-uveitis		
	N	No. of cataract	N	No. of cataract	HR (95% C.I.)
Wash-out period					
1 month	22523	639	22681	44	15.74 (11.59–21.30)
3 months	22450	566	22672	47	13.22 (9.82–17.80)
6 months	22388	504	22668	43	12.93 (9.47–17.64)

© 2024 Hsu AY et al. *JAMA Network Open*.

eTable 4. Risk of Cataract Exposure by Follow-up Duration of Different Aetiology of Uveitis and Anatomical Location

	No. of cataract		
	Uveitis	Non-uveitis	HR (95% C.I.)
**Year = 2017-2022**	N = 13684	N = 13684	
Cataract	438 (3.2)	30 (0.2)	15.96 (11.02–23.10)
Infantile, juvenile and presenile cataract	88 (0.6)	10 (0.1)	19.02 (7.73–46.84)
Traumatic cataract	75 (0.5)	10 (0.1)	15.40 (6.23–38.07)
Complicated cataract	116 (0.8)	10 (0.1)	63.89 (15.78–258.00)
Drug-induced Cataract	17 (0.1)	0 (0.0)	N/A
After-cataract (secondary cataract)	88 (0.6)	10 (0.1)	24.52 (9.00–66.78)
Cataracts unspecified	246 (1.8)	19 (0.1)	14.22 (8.92–22.67)
**Year = 2011-2016**	N = 8227	N = 8227	
Cataract	375 (4.6)	35 (0.4)	11.98 (8.48–16.94)
Infantile, juvenile and presenile cataract	81 (1.0)	10 (0.1)	14.91 (6.51–34.17)
Traumatic cataract	59 (0.7)	10 (0.1)	6.23 (3.19–12.18)
Complicated cataract	101 (1.2)	0 (0.0)	N/A
Drug-induced Cataract	22 (0.3)	10 (0.1)	24.46 (3.30–181.40)
After-cataract (secondary cataract)	93 (1.1)	10 (0.1)	10.29 (5.36–19.75)
Cataracts unspecified	232 (2.8)	17 (0.2)	15.28 (9.34–25.00)

N/A: Not applicable.

© 2024 Hsu AY et al. *JAMA Network Open*.

eTable 5. Risk of Cataract Exposure by Different Aetiology of Uveitis and Anatomical Location Uveitis

	Number of cataracts		
	Uveitis	Non-uveitis	HR (95% C.I.)
**Anterior**	N = 15727	N = 15727	
1 months	149	10	15.07 (7.95–28.59)
2 months	190	11	17.60 (9.59–32.32)
3 months	208	13	16.36 (9.34–28.65)
6 months	254	13	20.15 (11.53–35.10)
9 months	310	15	21.50 (12.80–36.10)
12 months	342	15	23.83 (14.20–39.90)
18 months	407	19	22.56 (14.24–35.70)
24 months	460	23	21.18 (13.93–32.10)
**Intermediate**	N = 2261	N = 2261	
1 months	10	10	9.84 (1.26–76.87)
2 months	13	10	12.84 (1.68–98.15)
3 months	23	10	11.50 (2.71–48.79)
6 months	30	10	7.58 (2.67–21.50)
9 months	40	10	10.23 (3.66–28.59)
12 months	46	10	11.85 (4.27–32.92)
18 months	52	10	13.47 (4.87–37.25)
24 months	66	10	11.59 (5.03–26.74)
**Posterior**	N = 215	N = 215	
1 months	0	0	N/A
2 months	0	0	N/A
3 months	0	0	N/A
6 months	10	0	N/A
9 months	10	10	2.18 (0.20–24.01)
12 months	10	10	3.27 (0.34–31.42)
18 months	10	10	5.52 (0.65–47.22)
24 months	10	10	6.69 (0.81–55.53)
**Pan uveitis**	N = 307	N = 307	
1 months	10	0	N/A
2 months	10	0	N/A
3 months	10	0	N/A
6 months	10	0	N/A
9 months	10	0	N/A
12 months	14	0	N/A
18 months	19	0	N/A
24 months	22	0	N/A

N/A: Not applicable.
Anterior: ICD-10-CM = H20
Intermediate: ICD-10-CM = H30, H31
Posterior: ICD-10-CM =H35.06
Pan uveitis ICD-10-CM = H44.11
Purulent endophthalmitis: ICD-10-CM = H44.003
Sympathetic uveitis: ICD-10-CM = H44.13
Ophthalmia nodosa: ICD-10-CM = H16.24
Secondary uveitis infectious: ICD-10-CM = H20.031, H20.039

© 2024 Hsu AY et al. *JAMA Network Open*.

eTable 6. Risk of Cataract Exposure by Different Aetiology of Uveitis and Anatomical Location Uveitis

	Uveitis		Non-uveitis		
	N	No. of cataract	N	No. of cataract	HR (95% C.I.)
Anatomical location of uveitis					
**Anterior**	15727	673	15727	42	17.37 (12.71–23.70)
**Intermediate**	2261	110	2261	10	13.07 (6.62–25.78)
**Posterior**	215	10	215	10	9.44 (1.18–75.58)
**Pan uveitis**	307	34	307	10	42.16 (5.77–308.20)
**Other etiologies**					
Purulent endophthalmitis	431	10	431	0	N/A
Sympathetic uveitis	139	10	139	0	N/A
Ophthalmia nodosa	10	N/A	10	N/A	N/A
Secondary uveitis infectious	23	10	23	0	N/A

N/A: Not applicable.
Anterior: ICD-10-CM = H20
Intermediate: ICD-10-CM = H30, H31
Posterior: ICD-10-CM =H35.06
Pan uveitis ICD-10-CM = H44.11
Purulent endophthalmitis: ICD-10-CM = H44.003
Sympathetic uveitis: ICD-10-CM = H44.13
Ophthalmia nodosa: ICD-10-CM = H16.24
Secondary uveitis infectious: ICD-10-CM = H20.031, H20.039

© 2024 Hsu AY et al. *JAMA Network Open*.

eTable 7. Stratification for Risk of Uveitis Exposed to Uveitis Compared With Nonuveitis

	Uveitis		Non-uveitis		
	N	No. of cataract	N	No. of cataract	HR (95% C.I.)
**Age**					
0–6	6198	184	6198	10	19.09 (10.10–36.00)
7–12	6823	321	6823	13	27.16 (15.59–47.20)
13–18	9582	298	9582	24	13.39 (8.84–20.30)
**Sex**					
Female	10281	416	10281	32	13.76 (9.60–19.71)
Male	12300	387	12300	35	11.97 (8.47–16.91)
**Race**					
Asian	617	27	617	10	13.80 (3.28–58.07)
Black	4408	106	4408	11	10.41 (5.60–19.36)
White	12357	476	12357	32	15.82 (11.05–22.60)
**Comorbidities**					
Asthma	1274	26	1274	10	6.65 (2.32–19.04)
Atopic dermatitis	431	10	431	10	5.17 (0.60–44.25)
Juvenile arthritis	1128	63	1128	10	59.31 (8.23–427.60)

If the patient's count is 1–10, the results indicate a count of 10.

© 2024 Hsu AY et al. *JAMA Network Open*.

eTable 8. Sensitivity Analysis for Risk of Cataract Exposed to Uveitis Compared With Nonuveitis

	Uveitis		Non-uveitis		
	N	No. of cataract	N	No. of cataract	HR (95% C.I.)
Intraocular surgery[a]					
No	20331	431	20331	32	14.49 (10.11–20.70)
Yes	651	52	651	10	11.07 (4.42–27.71)
Use of steroid eye drop[b]	3335	214	3335	10	29.51 (14.56–59.70)
Use of steroid eye drop†	3234	113	3330	10	45.27 (14.38–142.00)
Use of immunosuppressive agents[c]	7737	449	7737	19	26.52 (16.75–41.90)
Use of immunosuppressive agents†	7575	287	7732	14	24.35 (14.23–41.60)
Exclude trauma-related cataracts	22529	646	22529	47	14.81 (11.01–19.90)

[a]Intraocular surgery: Defined as performing the operation two years before the index date.
[b]Use of steroid eye drop: Defined as uveitis group with the use of steroid eye drop (ophthalmic product of prednisolone, dexamethasone, hydrocortisone) within three months after the index date.
[c]Use of immunosuppressive agents: Defined as uveitis group with the use of immunosuppressive agents (prednisolone, betamethasone, methylprednisolone, triamcinolone, dexamethasone, sulfasalazine, azathioprine, methotrexate, cyclosporine, cyclophosphamide, tacrolimus, leflunomide) within three months after the index date.
†Performed with a wash-out period of three months.
If the patient's count is 1–10, the results indicate a count of 10.

© 2024 Hsu AY et al. *JAMA Network Open*.

eTable 9. Risk of Cataract Exposed to Uveitis Compared With Nonuveitis by Steroid Eye Drop, Immunosuppressive Agents, C-Reactive Protein, and Leukocytes

	Uveitis		Non-uveitis		
	N	No. of cataract	N	No. of cataract	HR (95% C.I.)
[a]Steroid eye drop: ophthalmic product of prednisolone, dexamethasone, hydrocortisone					
Prednisolone	2781	165	2781	10	26.42 (12.39–56.20)
Dexamethasone	338	12	338	10	13.34 (1.74–102.60)
Hydrocortisone	10	0	10	0	N/A
[b]Immunosuppressive agents					
Prednisolone	4325	168	4325	11	17.97 (9.76–33.08)
Betamethasone	16	10	16	0	N/A
Methylprednisolone	214	10	214	10	8.19 (1.001–66.94)
Triamcinolone	230	13	230	10	13.67 (1.79–104.50)
Dexamethasone	709	20	709	10	7.28 (2.16–24.51)
Sulfasalazine	15	0	15	0	N/A
Azathioprine	29	0	29	0	N/A
Methotrexate	771	56	771	10	20.52 (6.42–65.62)
Cyclosporine	10	0	10	0	N/A
Cyclophosphamide	10	0	10	0	N/A
Tacrolimus	11	0	11	0	N/A
Leflunomide	10	0	10	0	N/A
[c]Without use of steroid eye drop	19427	600	19427	39	16.49 (11.92–22.70)
[d]Without use of immunosuppressive agents	14949	355	14949	21	17.69 (11.39–27.40)
C reactive protein [Mass/volume] in Serum, Plasma or Blood (mg/L)					
<10	717	30	717	10	6.06 (2.35–15.62)
≥10	743	39	743	10	13.80 (4.26–44.65)
Leukocytes [#/volume] in Blood (10*3/uL)					
<11	3221	107	3221	12	9.41 (5.18–17.09)
≥11	1115	37	1115	10	9.64 (3.44–27.04)

N/A: Not applicable.

If the patient's count is 1-10, the results indicate a count of 10.

[a]Use of steroid eye drop: Defined as uveitis group with the use of steroid eye drop (ophthalmic product of prednisolone, dexamethasone, hydrocortisone) within three months after the index date. To avoid drug interactions, the above-mentioned medications should be used exclusively without the concurrent use of medications from other classes.

[b]Use of immunosuppressive agents: Defined as uveitis group with the use of immunosuppressive agents (prednisolone, betamethasone, methylprednisolone, triamcinolone, dexamethasone, sulfasalazine, azathioprine,

© 2024 Hsu AY et al. *JAMA Network Open*.

methotrexate, cyclosporine, cyclophosphamide, tacrolimus, leflunomide) within three months after the index date. To avoid drug interactions, the above-mentioned medications should be used exclusively without the concurrent use of medications from other classes.

[c]Without use of steroid eye drop: Defined as uveitis group without use of steroid eye drop (ophthalmic product of prednisolone, dexamethasone, hydrocortisone) within three months after the index date

[d]Without use of immunosuppressive agents: Defined as uveitis group without use of immunosuppressive agents (prednisolone, betamethasone, methylprednisolone, triamcinolone, dexamethasone, sulfasalazine, azathioprine, methotrexate, cyclosporine, cyclophosphamide, tacrolimus, leflunomide) within three months after the index date

© 2024 Hsu AY et al. *JAMA Network Open*.

eTable 10. Stratification Analysis for Risk of Cataract Exposed to Uveitis Compared With Nonuveitis

	Uveitis		Non-uveitis		
	N	No. of cataract	N	No. of cataract	HR (95% C.I.)
[a]Steroidal immunosuppressive agents	6848	390	6848	74	5.62 (4.38–7.20)
[b]Absence of history of steroidal immunosuppressive agents	877	59	877	10	20.12 (6.31–64.17)
[c]Absence of history of autoimmune diseases	20272	724	20272	61	12.94 (9.96–16.79)
[d]History of autoimmune diseases	2412	80	2412	10	11.51 (5.32–24.92)

If the patient's count is 1–10, the results indicate a count of 10.
[a]**Steroidal immunosuppressive agents:** Defined as the use of immunosuppressive agents (prednisolone, dexamethasone, betamethasone, triamcinolone, methylprednisolone) within three months after the index date.
[b]**Absence of history of steroidal immunosuppressive agents:** Defined as the non-use of steroidal immunosuppressive agents (prednisolone, dexamethasone, betamethasone, triamcinolone, methylprednisolone) within three months after the index date.
[c]**Absence of history of autoimmune diseases:** Defined as non-diagnosis of autoimmune diseases within two years before the index date.
[d]**History of autoimmune diseases:** Defined as diagnosis of autoimmune diseases within two years before the index date

© 2024 Hsu AY et al. *JAMA Network Open*.

eTable 11. Positive Outcome Control, Negative Outcome Control Analysis

	Uveitis		Non-uveitis		
	N	No. of event	N	No. of event	HR (95% C.I.)
**Positive outcome control**					
Risk of steroid eye drop usage	20218	1582	20218	453	3.73 (3.36–4.14)
**Negative outcome control**					
Risk of osteoporosis	23,485	33	23,485	23	1.50 (0.88–2.56)

Osteoporosis: ICD-10-CM = M81.0
If the patient's count is 1-10, the results indicate a count of 10.
N/A: Not applicable.

© 2024 Hsu AY et al. *JAMA Network Open*.

eTable 12. Demographic Characteristics of Uveitis and Nonuveitis by Using the EMEA Network

	Before PSM				After PSM			
	Uveitis N = 1873	Non-uveitis N = 143316	p	Std diff.	Uveitis N = 1791	Non-uveitis N = 1791	p	Std diff.
Age	9.7 ± 5.3	5.9 ± 5.5	<0.001	0.704	9.7 ± 5.3	9.7 ± 5.3	0.965	0.001
Sex								
Female	906 (48.4)	69302 (48.4)	0.989	<0.001	861 (48.1)	854 (47.7)	0.815	0.008
Male	967 (51.6)	73987 (51.6)	0.998	<0.001	930 (51.9)	937 (52.3)	0.815	0.008
Unknown Gender	0 (0.0)	27 (0.0)	0.552	0.019	0 (0.0)	0 (0.0)	<0.001	<0.001
Race								
American Indian or Alaska Native	0 (0.0)	0 (0.0)	<0.001	<0.001	0 (0.0)	0 (0.0)	<0.001	<0.001
Asian	14 (0.7)	1532 (1.1)	0.178	0.034	13 (0.7)	14 (0.8)	0.847	0.006
Black or African American	10 (0.5)	253 (0.2)	<0.001	0.060	10 (0.6)	10 (0.6)	1	<0.001
Native Hawaiian or Other Pacific Islander	0 (0.0)	0 (0.0)	<0.001	<0.001	0 (0.0)	0 (0.0)	<0.001	<0.001
Unknown Race	1773 (94.7)	130395 (91.0)	<0.001	0.143	1695 (94.6)	1691 (94.4)	0.769	0.010
White	77 (4.1)	10493 (7.3)	<0.001	0.139	74 (4.1)	77 (4.3)	0.803	0.008
Other Race*	10 (0.5)	643 (0.4)	0.584	0.012	10 (0.6)	10 (0.6)	1	<0.001
Comorbidities								
Asthma	24 (1.3)	2101 (1.5)	0.509	0.016	24 (1.3)	22 (1.2)	0.767	0.010
Atopic dermatitis	10 (0.5)	2043 (1.4)	0.001	0.091	10 (0.6)	10 (0.6)	1	<0.001
RA	0 (0.0)	10 (0.0)	0.718	0.012	0 (0)	0 (0)	N/A	N/A
Other rheumatoid arthritis	10 (0.5)	18 (0.0)	<0.001	0.100	10 (0.6)	10 (0.6)	1	<0.001
Enteropathic arthropathies	0 (0.0)	10 (0.0)	0.718	0.012	0 (0)	0 (0)	<0.001	<0.001
Juvenile arthritis	63 (3.4)	66 (0.0)	<0.001	0.258	42 (2.3)	42 (2.3)	1	<0.001
Crohn's disease	10 (0.5)	63 (0.0)	<0.001	0.091	10 (0.6)	0 (0.0)	0.002	0.106
Ulcerative colitis	10 (0.5)	56 (0.0)	<0.001	0.093	10 (0.6)	10 (0.6)	1	<0.001
Celiac disease	10 (0.5)	93 (0.1)	<0.001	0.086	10 (0.6)	10 (0.6)	1	<0.001
Myopia	28 (1.5)	0 (0)	<0.001	0.174	0 (0)	0 (0)	N/A	N/A
Hypermetropia	37 (2.0)	0 (0)	<0.001	0.201	0 (0)	0 (0)	N/A	N/A
Medical utilization								
Ambulatory	840 (44.85)	40923 (28.55)	<0.001	0.343	767 (42.83)	762 (42.55)	0.866	0.006
Emergency	306 (16.34)	19499 (13.61)	0.001	0.077	291 (16.25)	291 (16.25)	1.000	<0.001
Inpatient Encounter	455 (24.29)	24014 (16.76)	<0.001	0.187	414 (23.12)	412 (23.00)	0.937	0.003
Laboratory								
C reactive protein [Mass/volume] in Serum, Plasma or Blood	15.8 ± 47.6	16.8 ± 34.4	0.756	0.024	16.5 ± 48.5	15.5 ± 29.6	0.859	0.024
Leukocytes [#/volume] in Blood	8.5 ± 3.3	12.3 ± 117.7	0.626	0.046	8.5 ± 3.3	8.7 ± 3.8	0.669	0.042

N/A: Not applicable.
Abbreviation: PSM, Propensity score matching; RA, Rheumatoid arthritis with rheumatoid factor; SD, standard deviation; Std diff., Standardized mean difference.
*Other race including mixed ethnicity

© 2024 Hsu AY et al. *JAMA Network Open*.

eTable 13. Risk of Cataract Exposed to Uveitis Compared With Nonuveitis by Using the EMEA Network

	No. of cataract		Free of cataract (%)		
	Uveitis N = 1791	Non-uveitis N = 1791	Uveitis	Non-uveitis	HR (95% C.I.)
All duration					
Cataract	32 (1.8)	10 (0.6)	94.10	99.48	7.23 (2.81–18.59)
Infantile, juvenile and presenile cataract	10 (0.6)	0 (0)	99.41	100.00	N/A
Traumatic cataract	10 (0.6)	0 (0)	99.73	100.00	N/A
Complicated cataract	13 (0.7)	10 (0.6)	97.75	99.67	4.80 (1.36–16.88)
Drug-induced Cataract	0 (0)	0 (0)	N/A	N/A	N/A
After-cataract (secondary cataract)	10 (0.6)	0 (0)	97.13	100.00	N/A
Cataracts unspecified	14 (0.8)	10 (0.6)	95.35	99.80	8.57 (1.94–37.81)

N/A: Not applicable.
If the patient's count is 1-10, the results indicate a count of 10.

eTable 14. Validation Analysis Using the Taiwan Insurance Research Database on the Risk of Cataract Among Patients With Uveitis

Variables	Event	PY	IR	Crude HR(95% CI)	p-value	adjusted HR (95% CI)	p-value
Uveitis							
No	172	53720	3.2	1.00(reference)	-	(1.00reference)	-
Yes	294	4195	70.09	20.1(16.65, 24.26)	<0.001	17.2(13.89, 21.30)	<0.001

© 2024 Hsu AY et al. *JAMA Network Open*.

eTable 15. Summary of Studies

Studies	Inclusion period, country	Sample size of uveitis patients (number of eyes)	Mean age or proportion of age groups involved among uveitis patients (years)	Medium onset of cataract after uveitis (months)	Outcome	Uveitis subtypes most involved with cataracts					Risk for increased cataract formation
						Ant.	Intermed.	Post	Pan	other	
Minkus et al. [2]	2001, United States	2190	<18 y/o (21%)	n/a	Cumulative incidence rate of 7.6% by one year and 36.6% by 10 years	n/a	n/a	n/a	n/a	Ant. and intermed. (28%)	Concurrent anterior uveitis and intermediate uveitis, Posterior synechiae, epiretinal membrane, higher dose of corticosteroid
Blum-Hareuveni et al. [9]	2017, United Kingdom	247	10.3 ± 0.4	96	37.2% developed cataract	48.8%	30.5%	6.7%	14%	n/a	Panuveitis, corticosteroid therapy, number of flare ups per year, development of cystoid macular edema and posterior synechiae
Kouwenberg et al. [14]	1997-2020, Unite	83	7.8	n/a	Increasing risk for cataract	100% (exclusively	n/a	n/a	n/a	n/a	n/a

© 2024 Hsu AY et al. *JAMA Network Open*.

	d States				surgery aHR = 2.90; *P* = .006	recruited)					
O'Rourke et al., [15]	2003 - 2016, Ireland	10	7.7	29.3 months	100% developed cataracts	n/a	n/a	n/a	n/a	n/a	Panuveitis, posterior synechiae, band keratopathy, glaucoma, CME
Papaliodis et al., [16]	1978-2010, United States	3923	n/a	n/a	507 eyes developed cataract (54/1000 eye-years)	n/a	n/a	n/a	n/a	n/a	Older age, higher anter chamber cell grade, prior incisional glaucoma surgery, band keratopathy, posterior synechia, elevated IOP
AlBloushi et al. [10]	1998-2017, Saudi Arabia	1582	38.6	26.4 months	18.3% eyes diagnosed with cataract	23.4%	n/a	n/a	n/a	n/a	Severity of anterior segment inflammation , keratic precipitate, posterior synechiae
Thorne et al., [17]	1984-2005, United States	75	7 (1-36)	55.2 months	0.04/eye-year	n/a	n/a	n/a	n/a	n/a	Posterior synechiae, severity of anterior chamber inflammation, topical corticosteroids

© 2024 Hsu AY et al. *JAMA Network Open*.

Al-Ani et al. [11]	2008-2020, New Zealand	562	38	117.6 months	22.1% developed cataract	96.5%	8.8%	n/a	2.3 %	n/a	n/a

© 2024 Hsu AY et al. *JAMA Network Open*.

eFigure 1. Brief Overview of the Study Design

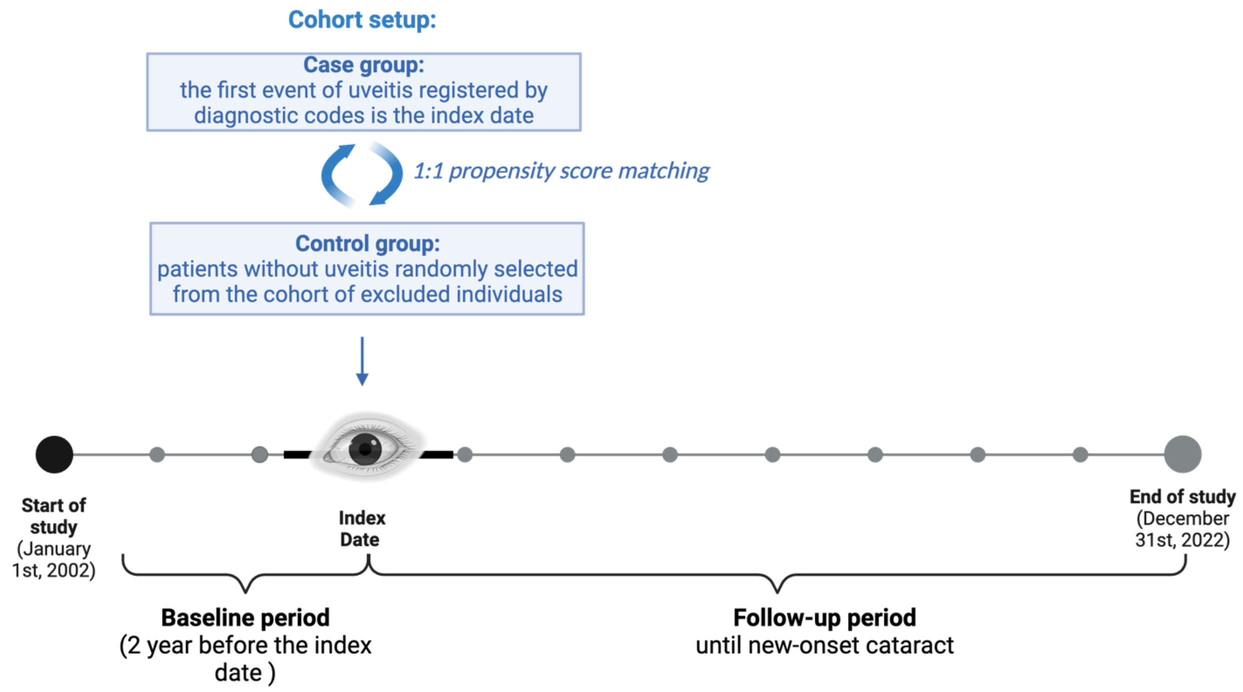

© 2024 Hsu AY et al. *JAMA Network Open*.

eFigure 2. Forest Plot of Stratification Analysis for Risk of Cataract

	Uveitis		Non-uveitis			
	N	No. of cataract	N	No. of cataract		HR (95% C.I.)
Age						
0–6	6198	184	6198	10		19.09 (10.10,36.00)
7–12	6823	321	6823	13		27.16 (15.59,47.20)
13–18	9582	298	9582	24		13.39 (8.84,20.30)
Sex						
Female	10281	416	10281	32		13.76 (9.60,19.71)
Male	12300	387	12300	35		11.97 (8.47,16.91)
Race						
White	12357	476	12357	32		15.82 (11.05,22.60)
Black	4408	106	4408	11		10.41 (5.60,19.36)
Asian	617	27	617	10		13.80 (3.28,58.07)
Comorbidities						
Asthma	1274	26	1274	10		6.65 (2.32,19.04)
Atopic dermatitis	431	10	431	10		5.17 (0.60,44.25)
Juvenile arthritis	1128	63	1128	10		59.31 (8.23,427.60)

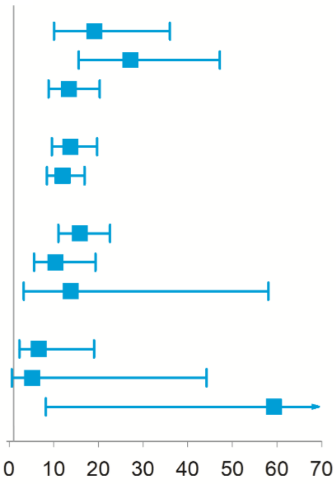

If the patient's count is 1–10, the results indicate a count of 10.

© 2024 Hsu AY et al. *JAMA Network Open*.

eFigure 3. Forest Plot of Sensitivity Analysis for Risk of Cataract Exposed to Uveitis Compared With Nonuveitis

	Uveitis		Non-uveitis		HR (95% C.I.)
	N	No. of cataract	N	No. of cataract	
Intraocular surgery					
No	20331	431	20331	32	14.49 (10.11,20.70)
Yes	651	52	651	10	11.07 (4.42,27.71)
Use of steroid eye drop	3335	214	3335	10	29.51 (14.56,59.70)
Use of steroid eye drop†	3234	113	3330	10	45.27 (14.38,142.00)
Use of immunosuppressive agents	7737	449	7737	19	26.52 (16.75,41.90)
Use of immunosuppressive agents†	7575	287	7732	14	24.35 (14.23,41.60)
Exclude trauma-related cataracts	22529	646	22529	47	14.81 (11.01,19.90)

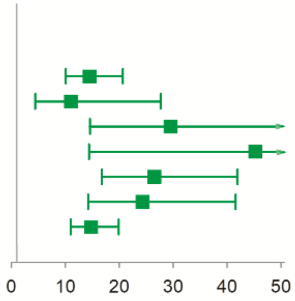

Intraocular surgery: Defined as performing the operation two years before the index date.
Use of steroid eye drop: Defined as uveitis group with the use of steroid eye drop.
Use of immunosuppressive agents: Defined as uveitis group with the use of immunosuppressive agents within three months after the index date.
Trauma-related cataracts: ICD-10-CM = H26.1.
†Performed with a wash-out period of three months.

© 2024 Hsu AY et al. *JAMA Network Open*.

eFigure 4. Kaplan-Meier Curves for Risk of Cataract Among Patients With Uveitis From the Taiwan Health Insurance Database

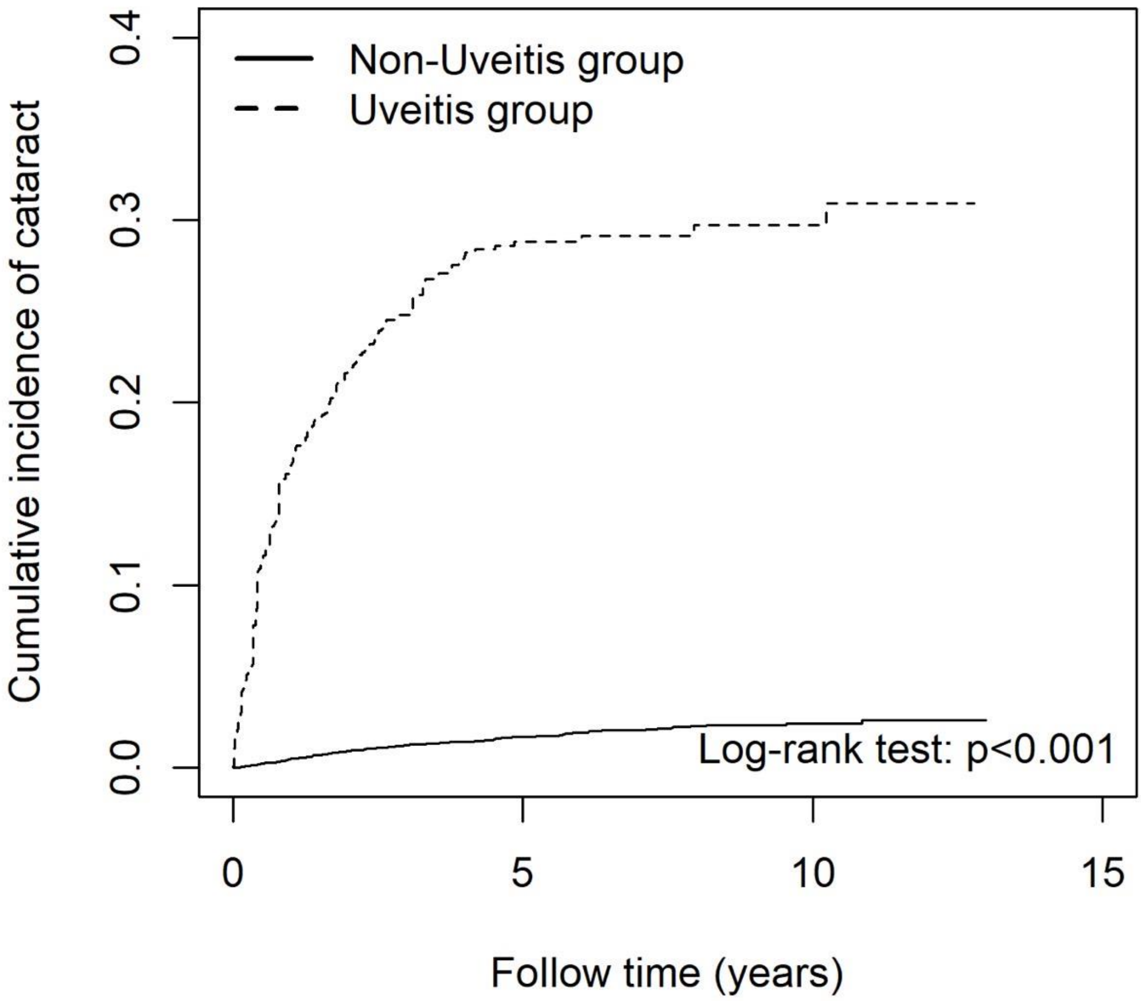

© 2024 Hsu AY et al. *JAMA Network Open*.

eFigure 5. Proposed Pathophysiology Contributing to Cataract Formation Among Pediatric Uveitis Eyes

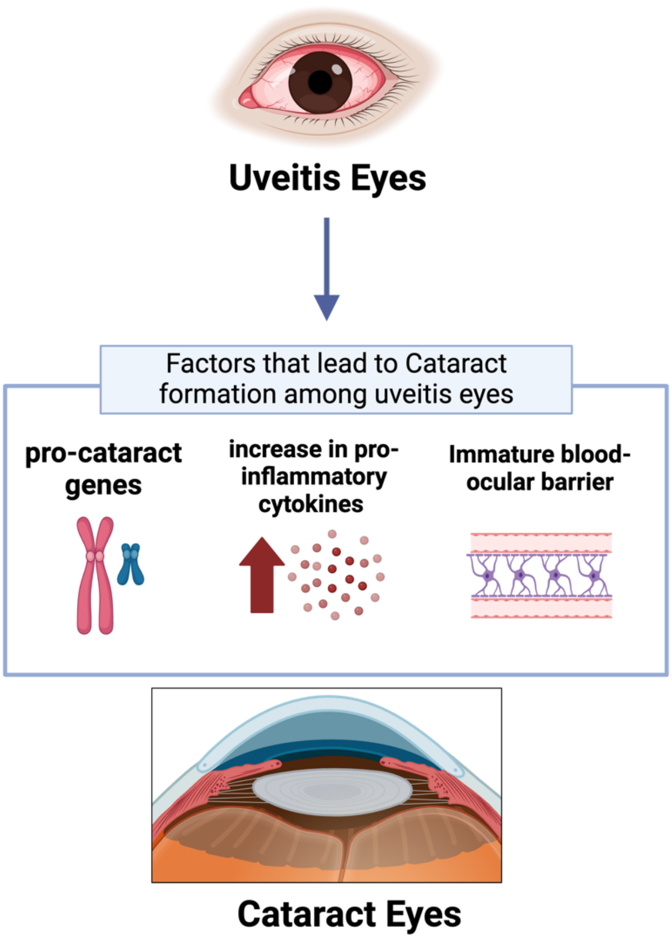

© 2024 Hsu AY et al. *JAMA Network Open*.
