# Supplementary material for: Cataract Development Among Pediatric Patients With Uveitis
Source: JAMA Netw Open. 2024 Jul 1;7(7):e2419366. doi: 10.1001/jamanetworkopen.2024.19366 (PMC11217876; doi:10.1001/jamanetworkopen.2024.19366)
Supplement: Supplement 2. — Data Sharing Statement [file jamanetwopen-e2419366-s002.pdf]

Data Sharing Statement

Hsu. Cataract Development Among Pediatric Patients With Uveitis. *JAMA Netw Open*. Published July 01, 2024. doi:10.1001/jamanetworkopen.2024.19366

Data
Data available: No
